# Prediction of Potential Cancer-Risk Regions Based on Transcriptome Data: Towards a Comprehensive View

**DOI:** 10.1371/journal.pone.0096320

**Published:** 2014-05-05

**Authors:** Arghavan Alisoltani, Hossein Fallahi, Mahdi Ebrahimi, Mansour Ebrahimi, Esmaeil Ebrahimie

**Affiliations:** 1 Department of Plant Breeding and Biotechnology, University of Shahrekord, Shahrekord, Iran; 2 Department of Biology, School of Sciences, Razi University, Kermanshah, Iran; 3 Department of Informatics, Saarland University, Saarbrucken, Germany; 4 Bioinformatics Research Group and Department of Biology, University of Qom, Qom, Iran; 5 School of Molecular and Biomedical Science, The University of Adelaide, Adelaide, Australia; University of North Carolina School of Medicine, United States of America

## Abstract

A novel integrative pipeline is presented for discovery of potential cancer-susceptibility regions (PCSRs) by calculating the number of altered genes at each chromosomal region, using expression microarray datasets of different human cancers (HCs). Our novel approach comprises primarily predicting PCSRs followed by identification of key genes in these regions to obtain potential regions harboring new cancer-associated variants. In addition to finding new cancer causal variants, another advantage in prediction of such risk regions is simultaneous study of different types of genomic variants in line with focusing on specific chromosomal regions. Using this pipeline we extracted numbers of regions with highly altered expression levels in cancer condition. Regulatory networks were also constructed for different types of cancers following the identification of altered mRNA and microRNAs. Interestingly, results showed that GAPDH, LIFR, ZEB2, mir-21, mir-30a, mir-141 and mir-200c, all located at PCSRs, are common altered factors in constructed networks. We found a number of clusters of altered mRNAs and miRNAs on predicted PCSRs (*e.g*.12p13.31) and their common regulators including KLF4 and SOX10. Large scale prediction of risk regions based on transcriptome data can open a window in comprehensive study of cancer risk factors and the other human diseases.

## Introduction

Alteration in mRNAs and miRNAs expression and the important role of a large number of these molecules have been studied in the initiation, progression and metastasis of many types of cancers [1], [2], [3]_ENREF_1. Changes in DNA methylation and transcription factor (TF) regulation, genomic copy number variation (CNV) [4], single nucleotide polymorphism (SNP) [5] and microsatellite alternation [6] as well as other chromosomal aberrations are characterized as major mechanisms of expression alternation in different human cancers (HCs).

Different methods including genome wide association studies (GWAS) have identified a large number of associated variants for different cancers [7], [8], [9]. For example, common variants on region 19p13 were found to be associated with ovarian cancer [10], CNVs at 6q13 and five risk loci at 21q21.3, 5p13.1, 21q22.3, 22q13.32 and 10q26.11 were directly linked to pancreatic cancer [4], [11]. In addition, new risk loci at 10q25.2, 6q22.2 and 6p21.32 were associated with lung cancer [12], and several risk loci at 9q31.2, 19q13.4 and 8q24 were shown to be associated with prostate cancer [13], [14], [15].

However, challenges in GWAS are finding causal variants and functional effects as well as interrelation of these variants in cancer. While previous genetic studies of cancer have predicted a large number of cancer-associated variants [8], [9], [10], [15], [16], identifying causal variants is major obstacle, because the known causal genetic variants are mostly located within non-coding regions or located at various physical distances from the gene they influence [17]. In addition, the employed linear modeling framework in GWAS often considers only one SNP at a time and ignores the effects of the other genotyped SNPs [5]. Therefore, the progression can be arduous from statistical association obtained through GWAS to inferred causality and functional consequences for cancer. Another challenge in large-scale genomics investigations is that some of these variants including microsatellites have been less studied compared to the other types (SNP and CNV). In addition, many of these studies are focused on one type of genomic variations in cancer; consequently, the impacts of other involved factors are neglected.

The common procedure employed in previous studies is detection of causal variants and searching for functional effects of these variants such as association of variants with expression quantitative trait loci (eQTLs) [17]. However, there is also a reverse strategy comprises prediction of potential cancer-risk regions shared across different types of cancers based on transcriptome expression data and then searching for causal variants. Identification of these regions assists in discovery of new variants as well as simultaneous study of different factors affecting gene expression by limiting assessments to specific chromosomal region. Here, we developed a pipeline which was comprised of PCSRs prediction using calculating the transcript-expression changes under cancer for each chromosomal region. We also extracted common altered mRNAs and microRNAs using microarray and expressed sequence tags (ESTs) data following by network analysis to achieve more insights about the predicted PCSRs. Using this pipeline, we predicted potential risk regions interacting with cluster of targets (mRNAs, miRNAs and/or TFs) unravelling potential-candidates for further genome association studies.

## Results

**Figure 1:**
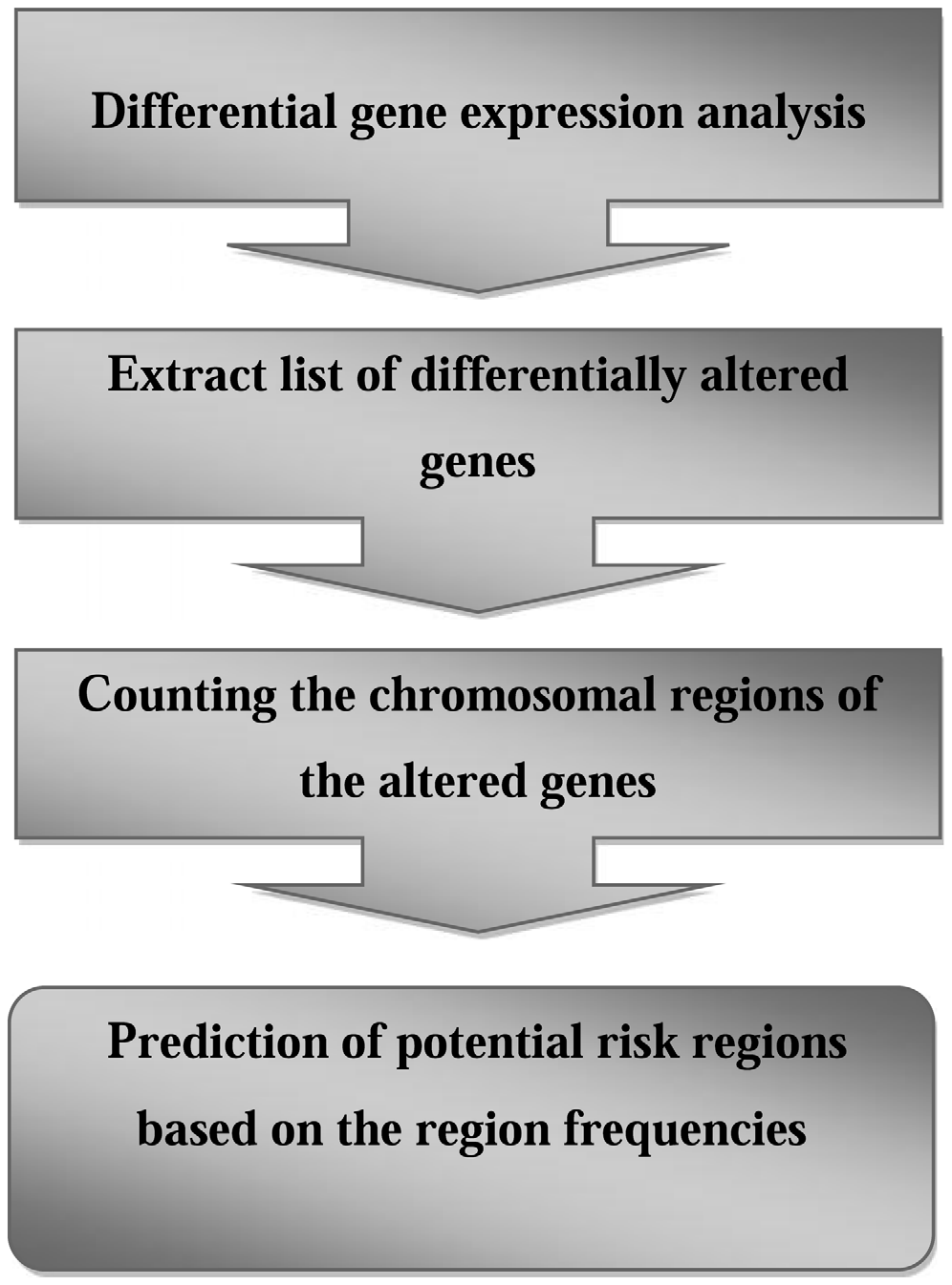
Analyzing workflow of prediction of potential risk regions. It comprises expression data analysis of different human cancers including breast, colorectal, endometrial, gastric, liver, lung, ovarian, pancreatic, prostate, testicular, bladder, intestine neuroendocrine, cervical and renal cancers as well as glioblastoma. This primary analysis followed by extraction of altered genes, count the chromosomal regions of altered genes and prediction of risk regions based on region frequency.

Gene expression data of several types of cancers were reanalyzed and the results were combined to predict common cancer-risk regions. Another aim of this study was to obtain insight into interrelation between PCSRs and altered mRNAs, miRNAs and their common regulators. An overview of the workflow is shown in Figure 1.

Results of transcript expression analyses for each cancer dataset including breast, colorectal, endometrial, gastric, liver, lung, ovarian, pancreatic, prostate, testicular, bladder, intestine neuroendocrine, cervical and renal cancers as well as glioblastoma are presented in Table S1. These extracted genes and miRNAs were then used for further analysis as outlined below.

### Prediction of Potential Cancer-Susceptibility Regions Using Microarray Datasets of Different Cancers

The percentage of region participation was calculated for each chromosome (chr) from microarray data (with 2-fold changes threshold) of 11 HCs. Details of procedure are described in materials and methods. For each chromosome, five regions covering the highest frequency of altered genes were recorded as potential PCSRs (Table 1). Results showed that among these PCSRs, two regions contain the highest number of over-expressed genes; chr1p31.2 (27.27%) and chr13q13.2 (20.45%) (Table 1, Columns 3 to 7). While in the case of down-expressed genes, the highest percentage was recorded for regions located at chr13q13 (15.53%) and 4q34.2 (15.15%).

**Table 1:** Predicted potential cancer-susceptibility regions (PSCRs) for probsets with at least 2 symmetrical fold changes using microarray datasets of 11 cancers including, breast, endometrial, ovarian, prostate, testicular, colorectal, liver, gastric, pancreatic, lung cancers and glioblastoma.

	Five Top Chromosomal Regions with highest percentage^a^ (%)									
Chromosome	Over-expressed					Down-expressed				
**1**	p11.2	p13.1	p21.1	p31.2	q32.3	p21.1	p31.3	p32.2	q31.1	q31.3
	8.33	7.01	11.48	27.27	6.27	7.18	6.68	8.50	8.23	7.98
**2**	p24.2	p25.1	p25.2	q22.2	q32.2	p23.1	q22.3	q24.1	q32.1	q32.3
	9.70	5.41	10.00	6.06	5.90	7.02	6.82	9.09	9.09	7.39
**3**	q12.1	q13.32	q25.2	q25.32	q25.33	p12.2	p24.1	q12.2	q13.11	q13.31
	5.68	9.09	7.51	5.61	8.46	9.09	7.79	8.48	8.33	8.91
**4**	p15.1	p15.31	q22.3	q28.3	q32.2	q23	q26	q28.1	q31.23	q34.2
	10.00	8.30	5.24	5.19	4.96	10.31	11.11	9.50	10.74	15.15
**5**	p14.1	p15.33	q11.1	q13.3	q14.2	p13.1	q11.1	q15	q21.3	q22.1
	4.55	5.13	9.09	4.32	12.12	12.52	9.09	8.67	11.26	10.23
**6**	p11.2	p22.2	q22.1	q22.31	q22.32	p24.1	q14.2	q22.31	q22.33	q24.2
	8.39	4.13	4.30	5.24	5.79	12.55	9.09	9.79	8.37	9.71
**7**	p12.1	p14.1	q21.3	q31.31	p15.3	q21.11	q21.12	q21.3	q31.1	q31.2
	6.49	6.71	6.20	6.93	7.27	10.20	8.65	7.54	6.88	7.95
**8**	q11.21	q13.2	q21.13	q24.12	q24.13	p11.22	p21.2	p22	q23.2	q24.11
	9.36	9.09	6.91	5.74	7.42	8.42	8.52	11.36	9.09	10.91
**9**	p13.1	q31.2	q21.2	q21.32	q22.2	p23	q21.11	q21.32	q31.3	q33.1
	8.08	5.23	4.55	8.48	8.64	12.73	6.55	6.97	9.09	8.66
**10**	p12.33	q21.1	q21.2	q23.33	q26.2	p12.33	q21.2	q23.2	q23.32	q23.33
	4.55	5.07	6.06	6.78	7.14	8.44	7.69	6.67	7.34	6.64
**11**	p11.12	p14.2	p15.2	q22.2	q24.1	p15.3	q14.2	q22.3	q23.2	q24.1
	14.55	6.29	4.96	13.13	4.68	9.53	7.64	6.22	6.94	9.35
**12**	p11.1	p11.21	p12.2	p12.3	p13.31	p12.3	q14.3	q21.2	q21.33	q24.21
	9.09	6.17	7.79	6.29	6.01	6.29	6.24	6.36	13.40	5.87
**13**	q13.2	q21.1	q21.33	q33.1	q33.3	q13.1	q13.3	q21.1	q22.2	q22.3
	20.45	13.64	4.81	4.32	4.55	8.68	10.10	9.09	7.69	15.53
**14**	q11.2	q21.1	q22.1	q22.2	q31.1	q13.1	q22.1	q22.2	q23.2	q31.3
	3.66	4.76	3.73	11.82	6.23	7.27	6.26	9.09	7.09	6.25
**15**	q12	q13.2	q21.3	q22.1	q26.1	q12	q13.2	q21.3	q22.1	q26.1
	4.09	4.24	4.20	5.45	5.18	4.09	4.24	4.20	5.45	5.18
**16**	p12.3	q11.2	q12.1	q21	q23.2	p13.12	q12.1	q12.2	q13	q23.2
	4.65	7.58	4.32	3.94	3.86	4.74	4.20	7.71	9.09	4.09
**17**	q11.2	q21.2	q21.33	q22	q24.3	p12	q11.1	q23.1	q24.2	q24.3
	2.72	3.44	2.90	3.70	5.94	5.50	11.11	4.40	7.20	6.49
**18**	p11.32	q11.2	q12.1	q21.32	q21.33	p11.23	q11.2	q12.3	q21.31	q22.1
	4.63	5.14	4.61	2.80	2.80	5.45	5.33	5.83	5.13	6.06
**19**	p12	p13.12	p13.3	q13.12	q13.41	p13.13	q12	q13.2	q13.31	q13.33
	6.02	2.98	2.44	2.60	3.90	7.27	4.11	2.66	2.75	3.12
**20**	p12.2	p12.3	q11.23	q13.2	q13.31	p11.21	p12.1	p12.3	q13.11	q13.13
	3.35	4.48	3.37	5.53	7.33	3.82	2.93	5.03	4.55	3.46
**21**	p11.2	q21.1	q22.11	q22.12	q22.2	q11.2	q21.3	q22.12	q22.13	q22.2
	4.04	3.68	3.21	3.68	3.36	4.28	5.92	3.90	4.36	4.35
**22**	q11.22	q11.23	q12.1	q13.31	q13.32	q11.22	q12.1	q13.1	q13.33	q12.2
	2.19	2.17	3.93	2.72	10.61	4.49	2.58	2.90	2.36	2.87
**X**	p22.32	q11.1	q21.32	q23	q26.2	p21.1	q13.2	q21.32	q22.1	q22.3
	6.82	5.45	9.09	4.86	8.82	7.07	8.86	12.12	7.34	7.77
**Y**	p11.32	q11.21	q11.222	q11.223		p11.31	q11.21	q11.222	q11.23	q12
	3.90	2.27	8.26	2.07		2.80	2.27	2.07	2.27	4.55

a Percentage: the fraction of altered probsets frequency for each region to the correspondence frequency of total probsets on microarray chip at the same region.

To test the reliability of the predicted PCSRs, the percentage of region participation in cancer was calculated with different threshold, where the frequencies of the first 200 probesets with highest fold changes were identified for each region (Table S2). While, a large number of these regions including 1q31.3, 2p25.2,3q25.2, 12p13.31 and 22q12.1 shared in both thresholds (Table 1 and Table S2), some regions were recorded as a PCSR for only one of these thresholds. For example 1p32.2 and 2q22.3 were identified for the 2-fold changes threshold, whereas, 1p22.3 and 2p12 were recorded for the highest fold changes (Table 1 and Table S2).

Percentage of chromosome participation was also calculated for 11 HCs, to identify which chromosome(s) is more involved in transcript expression changes (Table S3). Results showed that chr4 is harboring the highest number of genes altered in cancer (excluding prostate and gastric cancers) (Table S3). In contrast, chrY has the lowest number of genes expressed in cancer. A summary of chromosomal participation of 11 HCs shows significant differences as indicated by General Chi-squared test. Four top chromosomes harboring the most down-expressed genes were chrs 4, 5, 13 and X, whereas in the case of over-expressed genes the highest numbers of alteration were recorded for chrs 1, 7, 8 and 12 (Figure S1).

### Altered MRNAs Shared across Different Types of Cancers

Differentially expressed mRNAs with the highest fold changes in at least 6 HCs were selected as the common altered mRNAs (Table 2 and Table 3). These common altered mRNAs were classified into three different expression groups. Class I showed over-expression in majority of cancer types such as tubulin alpha 1b (TUBA1B) and glyceraldehyde-3-phosphate dehydrogenase (GAPDH) (Table 2), class II represented down-expression in most of HCs such as aspartoacylase (ASPA) and chemokine (C-X-C motif) ligand 12 (CXCL12) (Table 2), while the rests (Class III) showed a mixed expression patterns in different types of cancers such as protein kinase (cAMP-dependent, catalytic) inhibitor beta (PKIB) (Table 3).

**Table 2:** Common altered mRNAs (including class I and II) extracted from 11 human cancers using digital differential display (DDD), together with the available online microarray.

Gene symbol	location	PCSR	Cancer cell type																						Expression class
			Breast		Endometrial		Ovarian		Prostate		Testicular		Colorectal		Liver		Gastric		Pancreatic		Glioblastoma		Lung		
			M	D	M	D	M	D	M	D	M	D	M	D	M	D	M	D	M	D	M	D	M	D	
GAPDH	chr12p13.31	✓	↑		↑		↑	↑		↑	↑		↑	↑	↑	↑	↑	↑		↑			↑	↑	Class I
CKS2	chr9q22.2	✓	↑		↑		↑				↑		↑		↑		↑		↑				↑		Class I
CEP55	chr10q23.33	✓			↑		↑				↑		↑		↑		↑		↑				↑		Class I
UHRF1	chr19p13.3	✓	↑		↑		↑				↑		↑		↑		↑		↑		↑		↑		Class I
RRM2	chr2p25.1	✓	↑		↑		↑				↑		↑		↑		↑				↑	↑	↑		Class I
TUBA1B	chr12q13.12	-	↑		↑	↑	↑	↑		↑		↑	↑		↑	↑	↑	↑		↑		↑	↑	↑	Class I
THY1	chr11q23.3	-	↑		↑		↑		↑		↑		↑		↑		↑						↑		Class I
AURKA	chr20q13.2	✓			↑		↑				↑		↑		↑		↑		↑				↑		Class I
FLJ39632	chr14q11.2	✓			↑		↑		↑		↑		↑		↑		↑				↑		↑		Class I
FAM83D	chr20q11.23	✓	↑		↑		↑				↑		↑		↑		↑		↑				↑		Class I
MAD2L1	chr4q27	✓	↑		↑		↑				↑		↑		↑		↑				↑		↑		Class I
NEK2	chr1q32.3	✓			↑		↑				↑		↑		↑		↑				↑		↑		Class I
ASPA	chr17p13.32	-	↓		↓		↓		↓				↓		↓		↓						↓		Class II
CXCL12	chr10q11.21	-	↓		↓		↓				↓		↓		↓		↓				↓		↓		Class II
PTGDS	chr9q34.3	-	↓				↓				↓		↓		↓								↓		Class II
MALAT1	chr11q13.1	-	↓	↓	↓		↓		↓		↓	↓	↓		↓	↓	↓		↓	↓	↓		↓	↓	Class II

Abbreviations: PCSR, Potential Cancer-Susceptibility Region; M, Microarray; D, Digital Differential Display.

Symbols: ↑, over-expression; ↓, down-expression; ✓, risk region.

**Table 3:** Common altered mRNAs (including class III) extracted from 11 human cancers using digital differential display (DDD), together with the available online microarray.

Gene symbol	location	PCSR	Cancer cell type																						Expression class
			Breast		Endometrial		Ovarian		Prostate		Testicular		Colorectal		Liver		Gastric		Pancreatic		Glioblastoma		Lung		
			M	D	M	D	M	D	M	D	M	D	M	D	M	D	M	D	M	D	M	D	M	D	
ZEB2	chr2q22.3	✓	↓		↓		↓		↓		↓		↓				↑				↓		↓		Class III
ZEB1	chr10p11.22	-			↓		↓		↑		↓		↓		↑		↑				↑		↓		Class III
KRT8	chr12q13.13	-	↑		↑	↑			↑	↑	↑		↓	↑	↓	↑		↑		↑		↑	↑	↑	Class III
ANXA2	chr15q22.2	✓			↑	↑	↓			↑	↓				↑	↑	↑		↑	↑		↑			Class III
IGF2BP3	chr7p11	✓			↑		↑		↑		↑		↑		↑		↑		↑		↓		↑		Class III
DDX5	chr17q23.3	-			↓	↓	↓			↓	↑	↓	↓									↓	↓	↓	Class III
CD24	chryq11.22	✓	↑		↑	↑	↑		↑	↓	↑	↑		↓	↑		↓					↑	↑		Class III
TOP2A	chr17q21.2	✓	↓		↑		↑				↑		↑		↑		↑		↑		↑		↑		Class III
DCN	chr12q21.33	✓			↓		↓		↓		↓		↓		↓			↓	↑			↓	↓	↓	Class III
CENPF	chr1q41	-	↑		↑		↑				↑		↑		↑		↑				↑		↑		Class III
LIFR	chr5p13.1	✓	↓		↓		↓				↑		↓		↓		↓						↓		Class III
ABCA8	chr17q24.2	✓	↓		↓		↓		↑		↓		↓		↓		↓						↓		Class III
COL11A1	chr1p21.1	✓	↑		↑		↓				↑		↑		↑		↑		↑				↑		Class III
RSAD2	chr2p25.2	✓	↑		↑		↑				↑		↓				↓		↑		↑				Class III
Cd36	chr7q21.11	✓	↓		↓		↓		↑				↓				↓				↑				Class III
PCDH7	chr4p15.1	✓	↑		↓		↑		↑		↑		↓						↑				↑		Class III
PKIB	chr6q22.31	✓	↑		↓				↓		↑		↓		↑		↓		↓		↓		↑		Class III
EDNRB	chr13q22.3	✓	↑		↑		↑		↑		↑		↑		↑						↓		↑		Class III
SULF1	chr8q13.1	✓	↑		↑		↓				↓		↑		↑		↑		↑		↑		↑		Class III
DIO2	chr14q31.1	✓	↑				↑		↑		↓		↑				↑		↑				↑		Class III
TMC5	chr16p12.3	✓	↑		↑		↑		↑		↓				↑				↑				↓		Class III
HS6ST2	chrXq26.2	✓			↓		↓		↓		↑		↑		↑		↑		↓				↑		Class III
PTGS2	chr1q31.1	✓	↓				↓				↓				↓				↑				↓		Class III
GPM6A	chr4q34.2	✓	↓		↓		↓		↓		↑		↓		↓						↑		↓		Class III
C7	chr5p13.1	✓	↓		↓	↓	↓		↓			↓	↓		↓						↑		↓		Class III
MT1M	chr16q12.2	✓	↓						↓		↑		↓		↓		↓				↑		↓		Class III

Abbreviations: PCSR, Potential Cancer-Susceptibility Region; M, Microarray; D, Digital Differential Display.

Symbols: ↑, over-expression; ↓, down-expression; ✓, risk region.

Interestingly, a number of common altered mRNAs are located on the predicted PCSRs (Column 3 of Table 2 and Table 3). For example, GAPDH at 12p13.31(as a predicted PCSR) showed over-expression in all of HCs (Table2). CKS2 (chr9q22.2), CEP55(chr10q23.33), UHRF1 (chr19p13.3), RRM2 (chr2p25.1), AURKA (chr20q13.2), FLJ39632 (chr14q11.2), FAM83D (chr20q11.23), NEK2 (chr1q32.3) and MAD2L (chr4q27) were all located on PCSRs and showed over-expression in the 9, 8, 10, 9, 8, 9, 9, 8 and 9 types of cancers, respectively (Table 2 and Table 3). In contrast, DCN (chr12q21.33), LIFR (chr5p13.1), ABCA8 (chr17q24.2), C7 (chr5p13.1) and ZEB2 (chr2q22.3) on predicted PCSRs were down-expressed in 9, 7, 8, 8 and 8 cancers, respectively (Table 2 and Table 3). The rest of altered genes on PCSRs exhibited both down and over-expression patterns (Table 3).

### Altered MiRNAs Shared across Different Cancers

Several types of miRNAs (such as miR-93, mir-182, mir-196b and mir-1274b) exhibited over-expression in majority of cancers (Table 3). A number of miRNAs (such as miR-30a and mir-30c-2) were down-expressed in various HCs, whereas, many other miRNAs exhibited a mixed pattern of expression (Table 4).

**Table 4:** List of common differentially expressed MicroRNAs in 15 different cancers. A numbers of these genes are located on predicted cancer susceptibility regions.

MicroRNA	Cancer cell type															location	PCSR
	Br	Ce	En	Ov	Pr	Te	Re	Co	Ga	Li	Pn	Gl	Ln	In	Bl		
hsa-mir-200c		↑		↑	↑		↓								↑	12p13.31	✓
hsa-mir-141				↑	↑		↓								↑	12p13.31	✓
hsa-mir-106a				↑	↑		↓								↑	Xq26.2	✓
hsa-mir-20b				↑	↑		↓								↑	Xq26.2	✓
hsa-mir-30c-2	↓	↓	↓	↓		↓	↓	↓					↓			6q13	✓
hsa-mir-30a		↓					↓								↓	6q13	✓
hsa-mir-500		↓					↓							↑	↑	Xp11.23	-
hsa-mir-532		↓					↓							↑		Xp11.23	-
hsa-mir-501		↓					↓								↑	Xp11.23	-
hsa-mir-502		↓					↓							↑		Xp11.23	-
hsa-mir-362		↓					↓								↑	Xp11.23	-
hsa-mir-432		↓					↓									14q32.2	✓
hsa-mir-770						↓		↓		↓						14q32.2	✓
hsa-mir-127		↓					↓								↓	14q32.2	✓
hsa-mir-379		↓					↓									14q32.31	-
hsa-mir-382		↓					↓								↓	14q32.31	-
hsa-mir-134		↓					↓								↓	14q32.31	-
hsa-mir-27b		↓					↓							↑		9q22.32	-
hsa-let-7d			↓	↓		↑	↓						↓		↑	9q22.32	-
hsa-mir-23b		↓					↓									9q22.32	-
hsa-mir-99b		↓			↓		↓								↑	19q13.41	✓
hsa-mir-125a		↓			↓		↓								↑	19q13.41	✓
hsa-mir-424		↓					↓									Xq26.3	-
hsa-mir-106b				↑	↑		↑							↑	↑	7q22.1	-
hsa-mir-93				↑	↑		↑								↑	7q22.1	-
hsa-mir-149		↓					↓								↓↔↑	2q37.3	-
hsa-mir-200b				↑	↑		↓								↑	1p36.33	-
hsa-mir-21	↑			↑	↑	↓	↑		↓						↑	17q23.1	✓
hsa-mir-126		↑		↑												9q34.3	-
hsa-mir-214	↑	↑	↓			↓	↓			↓		↓			↓	1q24.3	✓
hsa-mir-101-1			↓	↓		↑					↑	↑	↓	↑		1p31.3	✓
hsa-miR-182				↑	↑									↑	↑	7q32.2	-
mir155HG (BI	↑		↓	↑		↓			↓	↑		↓	↓			21q21.3	✓
hsa-mir-30e		↓					↓							↑	↑	1p34.2	-
hsa-mir-422a		↓					↓							↓	↑	15q22.31	-
hsa-mir-132				↓			↓								↓	17p13.3	-
hsa-mir-205		↓		↓↔↑	↓										↓↔↑	1q32.2	-
hsa-mir-375		↑			↑											2q35	-
hsa-mir-1274b		↑			↑										↑	19q13.43	-
hsa-mir-361		↓					↓							↑	↓↔↑	Xq21.2	-

Abbreviations: Br, Breast; Ce, Cervical; En, Endometrial; Ov, Ovarian; Pr, Prostate; Te, Testicular; Re, Renal; Co, Colorectal; Ga, Gastric; Li, Liver; Pn, Pancreatic; Gl, Glioblastoma; Ln, Lung; In, Intestinal Neuroendocrine; Bl, Bladder; PCSR, Potential Cancer-Susceptibility Region.

Symbols: ↑, over-expression; ↓, down-expression; ✓, risk region.

The chromosomal locations were determined for common altered miRNAs. Interestingly, miRNAs located on the same region showed co-expression in some cancers, such as a cluster at 19q13.41 (including mir-99b and -125a). This cluster (19q13.41) was down-expressed in cervical, prostate and renal cancers. In contrast, the same cluster was over-expressed in bladder cancer. Another co-expressed cluster was observed at 12p13.31 (mir-141and mir-200c), which showed over-expression in ovarian, prostate and bladder cancers, and conversely, it were down-expressed in renal cancer (Table 4). The rest of co-expressed clusters were listed for regions at 6q13 (including mir-30a and mir-30c-2), Xp11.23 (including mir-362, mir-500, mir-501, mir-502 and mir-532), 14q32.2 (including mir-134, mir-379 and mir-382), 14q32.31 (including mir-127, mir-432 and mir-770), 9q22.32 (including let-7d, mir-23b and mir-27b) and 7q22.1 (including mir-93 and mir-106b) (Table 3). Five out of nine miRNA co-expressed clusters listed above are located at predicted PCSRs including 6q13, 12p13.31, 14q32.2, 19q13.41 and Xq26.2 (Table 4).

### Interaction within and between Common Altered MRNAs and MiRNAs Revealed by Network Analysis

Four separate networks were constructed including a network for common altered mRNAs (with 409 entities and 1288 relations) (Figure S2), a network for common altered mRNAs located on the different predicted PCSRs (with 383 entities and 1121 relations) (Figure S3), a network of common altered miRNAs (with 322 entities and 1041 relations) (Figure S4) and a network for common altered miRNAs located on the different PCSRs (with123 entities and 409 relations) (Figure S5). In addition, a combined network was constructed by integration of altered mRNAs and miRNAs data, which has 667 entities and 2482 relations (Figure S6). Various type of transcription factors, protein kinases, small molecules, mRNAs and miRNAs serve as either validated or putative regulators in these networks. Additional details of each network including number of imported genes and biological processes presented in Table S4.

We identified networks with similar biological processes, such as cellular process, biological regulation, metabolic process, multicellular organismal process, developmental process and response to stimulus (Table S4 Column 5). These shared processes imply existence of common genes and miRNAs across different constructed networks as listed in Table S5. For example, Zinc finger E-box binding homeobox 2 (ZEB2), DEAD (Asp-Glu-Ala-Asp) box helicase 5 (DDX5) and leukemia inhibitory factor receptor alpha (LIFR) were shared between both constructed networks of common altered mRNAs and miRNAs (Table S5). Among common altered miRNAs, mir-21, mir-30a, mir-141 and mir-200c were shared across all of the four constructed networks (Table S5).

**Figure 2:**
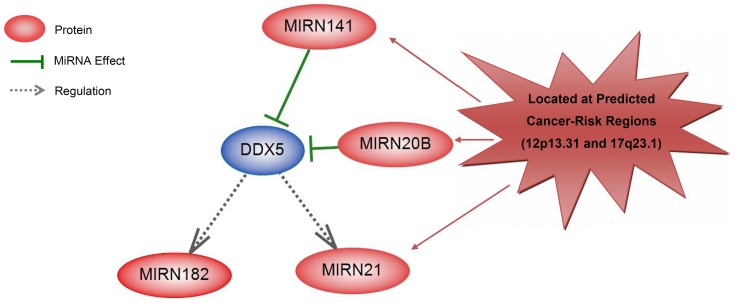
Subnetwork center on DDX5 derived from network of common altered variants in different cancers. Network is including mir-21, mir-182, -mir20b and mir-141. Network was constructed using pathway studio 9 software. Network was assembled based on bioinformatics and literature, combined with biological interpretation of the microarray data and enriched Gene Ontology functional groups. Red: over-regulated entities in most of cancers. Blue: down-regulated entities in most of cancers. 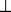
represents negative-regulated.

The most frequent subnetwork observed in these networks was centered on DDX5 (Figure 2). This subnetwork comprises 5 entities including DDX5, mir-20b, mir-21, mir-141 and mir-182. DDX5 is negatively regulated by mir-20b and mir-141, while DDX5 itself regulates mir-21 and mir-182. Down-expression of DDX5 was observed in 7 types of HCs, while, mir-20b, mir-21, mir-141 and mir-182 over-expressed in 3, 5, 3 and 4 HCs, respectively (Table 3 and Table 4). It suggests the negative interrelation between DDX5 and these four miRNAs.

**Figure 3:**
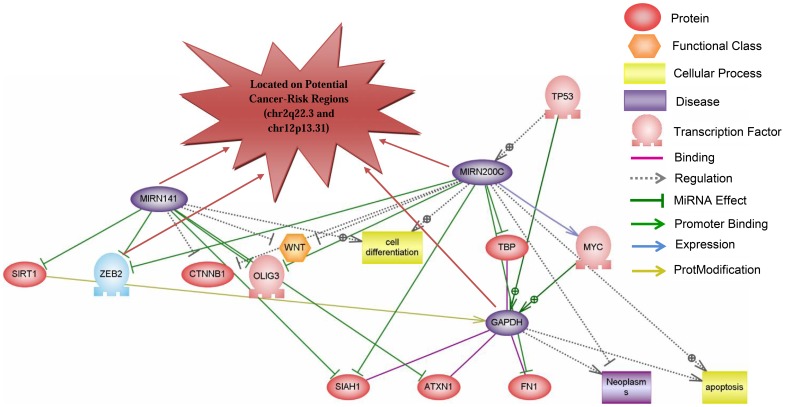
Network of common altered variants in different cancers including mir-200c, mir-141, and GAPDH at 12p13.3. Network was constructed using pathway studio 9 software. Shortest path algorithm was applied to construct network. Network was assembled based on bioinformatics and literature, combined with biological interpretation of the microarray data and enriched Gene Ontology functional groups. Purple: over-regulated entities in most of cancers Blue: down-regulated entities in most of cancers. O-vertex represent TFs, 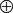
represents positive-regulated, and 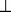
represents negative-regulated.

Another subnetwork was constructed based on mir-141, mir-200c, and GAPDH, which all located on predicted PCSRs at 12p13.31 (Figure 3). This network comprises of 17 entities and 29 relations (Figure 3). Thirteen downstream targets were observed for mir-141, mir-200c, and GAPDH. For example, mir-141 and, mir-200c, which were over-expressed in 3 HCs (shown as purple in the Figure 3), have miRNA effects on ZEB2 (with down-expression in 7 HCs). Interestingly, these altered RNAs including mir-141, mir-200c and GAPDH (at 12p13.31) and also ZEB2 (at 2q22.3) are all located at predicted PCSRs. In the case of upstream nodes, TP53 and MYC were observed as upstream regulators of mir-200c and GAPDH (Figure 3). TP53 is common positive regulator for both mir-200c and GAPDH, but MYC is only regulating GPADH (Figure 3).

### Promoter Analysis of Altered MRNAs and MiRNAs across Different Cancers

Promoters of over-expressed and down-expressed mRNAs and miRNAs were individually analyzed across different cancers. A list of common transcription factors for each set of down-expressed and over-expressed mRNAs are provided in the Tables S6 and S7, respectively. Among 18 common predicted TFs for over-expressed mRNAs, Kruppel-Like Factor 4 (KLF4) located at PCSRs was found to be down-expressed in 7 types of cancers (Table S6). While, from total 13 common regulators predicted for down-expressed mRNAs, 6 regulators are located on PCSRs. Among these 6 regulators RAR-related orphan receptor A (RORA) was down-expressed in 8 types of cancers (Except that Glioblastoma with over-expression and no significant expression in prostate and gastric cancers) (Table S7).

Common regulators were also predicted for cluster of altered miRNAs on the same region (Table S8). For example, GATA2, GATA3, ETS1, MZF1_1-4, SOX10, YY1, ZNF354C and SPI1 were predicted for miRNAs located on cluster at Xp11.23 (Table S8). In total, 22 common regulators were predicted for different clusters of miRNAs which eight of them are located at PCSRs including YY1, SPIB, SOX10, NFIC, NR4A2, FOXD1, NFATC2 and HOXA5 (Table S9). Interestingly, GATA2 was predicted for both down-expressed mRNAs and altered miRNAs.

## Discussion

An effective pipeline was developed to predict PCSRs using microarray datasets of different cancer studies. Two different thresholds were applied to predict PCSRs including probsets with at least 2-fold changes and first 200 probsets with the highest fold changes. Most of the predicted PCSRs on each chromosome were similar in both applied thresholds, which confirm the reliability of these PCSRs.

In addition to this confirmation, based on literature review we found the presence of several important cancer-associated variants on our predicted PCSRs. These variants have been reported previously for pancreatic [4], [11] (6q13, 21q21.3, 5p13.1, 21q22.3 and 22q13.32), lung [12] (6p21.32), prostate [13], [14], [15] (9q31.2, 19q13.4, 8q24 and 17q21-q22), ovarian [10] (19p13), breast [18] (8q24, 12p13 and 20q13) and colorectal cancer [19] (11q23, 8q24 and 18q21). Our findings in agreement with these studies identified region 8q24 as a risk region in variety of HCs [8], [14], [19], [20], [21], which shows involvement of some of risk regions in several types of cancers rather than a specific cancer. Moreover, some of the predicted PCSRs in this study were reported in other types of human diseases including herpes simplex virus type 1 [22] (21q), polycystic ovary syndrome [23] (9q33.3), Type 1 diabetes and Rheumatoid arthritis [24] (both located on 18p11). This similarity might indicate the efficiency of our approach in prediction the risk regions associated with different human diseases besides cancer.

We also found that eight chromosomes harbor the most altered genes in different types of cancer including chromosomes 1, 4, 5, 7, 8, 12, 13 and X. Interestingly, chromosomes 1, 4 and 13 were also recorded as the chromosomes with the highest percentage of predicted PCSRs, which suggests the important role of these chromosomes in cancer biology. Based on these results and those previously reported on chromosomes abnormality [7], [25], [26], [27], it can be concluded that our pipeline is able to predict risk regions as well as risk chromosomes in a variety of diseases including cancer. This pipeline can also be applied to the fast growing (but still limited number of) RNA-seq datasets in future studies.

Network analysis indicates that DDX5, LIFR, ZEB2, mir-21, mir-27b, mir-30a, mir-141, mir-182 and mir-200c were shared across different constructed networks, indicting their crucial role in cancer biology and progression, which has been reported previously [28], [29], [30]. For example, the potential clinical utility of DDX5 and its associated miRNAs (mir-21 and mir-182) are suggested as therapeutic target in breast cancer [29], [31]. In addition, clinical application of different miRNAs in cancer such as let-7, mir-21and mir-122 are discussed in recent study of Nana-Sinkam and Croce [28].

Because miRNAs do not function in isolation [28], we analyzed the cluster of miRNAs on same regions to understand the relative contribution of multiple miRNAs rather than individual miRNA. Co-expression of different miRNA implies the presence of common transcription regulators and/or common causal variants for these regions. It is also previously reported that common modules on the promoters can cause co-expression of the genes [32].

We found that different common regulators for altered mRNAs and miRNAs including, KLF4 (at 9q31.2) and RORA (15q22.2) were on the predicted PCSRs. These two TFs mediate a set of cell-cycle genes and exhibits both oncogenic and tumor suppressive functions [33], [34]. Interestingly, down-expression of mir-30c-2 (at 6q13) as well as over-expression of GATA3 was observed across different types of HCs in this study, which confirm regulation of mir-30c-2 through GATA3. Bockhorn and collogues recently demonstrated that mir-30c is transcriptionally regulated with GATA3 [35].

Presence of another level of interrelation between cancer-risk regions was suggested, where mRNAs and their common regulators at different PCSRs interact with each other as well as their targets. The subnetwork centered on DDX5 with total 5 nodes and 4 relations (Figure 2) and the subnetwork of GAPDH, miR-141 and mir-200c confirm such interactions (Figure 3). In these subnetworks, different RNAs are located on PCSRs including GAPDH, ZEB2, mir-20b, mir-21, mir-141 and mir-200c supporting the important effects of these RNAs and their regions in cancer.

Subnetwork centered on DDX5 is shared across networks constructed for altered mRNAs and miRNAs in different cancers. RNA helicase DDX5 (also known as p68) is involved in RNA metabolism and serves as a transcriptional co-regulator and has been reported as regulator of mir-182 in breast cancer [29]. Significant association has been also reported between DDX5 rs1991401 (OP = 7.90×10−5) and malignant peripheral nerve sheath tumor [36]. Our results showed that up regulation of mir-20b and mir-141 down regulates DDX5.

Second subnetwork (Figure 3) contained GAPDH, mir-141 and mir-200c that are located at 12p13.31 as predicted PCSRs. Amplification of 12p13 region was observed in breast cancer [37], T cell lymphomas and lymphocytic leukemia [38], [39], causing over-expression of GAPDH, mir-141 and -200c. Upstream regulators can involve in up-regulation of these RNAs and a positive effect has been reported for TP53 located on the upstream region of GAPDH [40]. In addition, Yoshihara et al [41] reported some sporadic ovarian cancer-unique CNVs at 12p13.31. In general, these reports in combination with our *in silico* findings indicate the crucial role of 12p13.31 in HCs.

Interestingly, some other common RNAs between cancers in this report, are observed in prior studies of tumors and other diseases [16], [42]. For example, presence of synonymous SNP (rs12948217) affecting the exonic splicing enhancers site nearby ASPA has been reported for neurodegenerative disease [43]. Loss of regions including 14q32.2 (location of mir-127, mir-432 and mir-770) and 14q32.31 (mir-134, mir-379, and mir-382) were reported in previous studies of renal cancer and osteosarcoma [16], [44]. In our study, mirRNAs located at 14q32.2 and 14q32.31 showed down-expression in several cancers, implying down-expression of miRNAs following chromosome loss in these regions.

In conclusion, predicted PCSRs in the current study opens new avenue in further genome association studies for finding different types of cancer-causal variants. Since multiple variations accumulated in a gene or a cluster of genes may all contribute to the phenotype, studying different types of variations or regulatory mechanisms over a gene, cluster of genes or specific region might be a useful tool for improving association detection. The identified common altered RNAs at PCSRs in our constructed networks have great potential to be used for finding associated SNPs, CNVs and/or SSRs near these genes. In addition, these results suggest the potential of novel regulator-based (rather than gene-based) cancer therapy in order to restore the disrupted cluster of mRNAs and/or miRNAs. In general, our pipeline can be effectively used to predict cancer-risk regions and cancer-risk chromosomes.

## Methods

### Expression Data Analysis

Raw CEL expression data for different HCs were obtained from Gene Expression Omnibus (GEO) database (Table S10). The RMA (Robust Multichip Average) algorithm was first applied to the microarray raw data to obtain normalized data using Expression Console software (Affymetrix, CA, USA). Data were then analyzed using FlexArray software (http://genomequebec.mcgill.ca/FlexArray/). Differential gene expression pattern for each experiment (cancer vs. normal) was evaluated using empirical Bayes test (a moderated t test) (p<0.05). Genes exhibiting at least 2-fold changes in gene expression and 1.5 fold changes in miRNA expression were selected for further analysis. Also, 1.2-fold change was considered to trace common altered mRNAs and miRNAs in different cancers.

The digital differential display (DDD) tool (http://www.ncbi.nlm.nih.gov/UniGene/ ddd.cgi) was used to screen the cancer-related genes in different HCs. EST libraries selected for DDD comparisons of different tissues (cancer vs. normal) are listed in Table S11. Pools A and B were assigned for normal and cancerous libraries in each cancer, respectively. The output provided a numerical value in each pool denoting the fraction of sequences within the pool that mapped to the UniGene cluster. Statistically significant hits (Fisher's exact test) showing >10-fold differences were compiled, and a preliminary database was created. Fold differences were calculated by using the ratio of pool B/pool A, according to previously described method [45].

Among probsets with highest fold changes, common altered mRNAs and miRNAs (at least in 6 out of 11 HCs) were extracted using DDD tools together with microarray datasets. These common altered RNAs afterward used for network constructions.

### Detecting of Shared-Cancer Susceptibility Regions

The numbers of differentially expressed genes were counted for each region (as frequency of the region) using an in-house developed python script (The python script is available in Script S1). The frequency of region involved in expression was calculated for probsets with at least 2-symmetrical fold changes (Table S12) and 200 first probsets with the highest fold changes (Table S13). Next for each region, percentage of region participation in differentially expressed probsets in all 11 types of HCs was calculated using following equations:
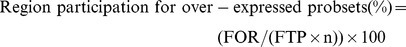
Where FOR is the frequency of region for over-expressed probsets (summation of 11 HCs), n is the number of cancers (here is 11) and FTP is frequency of region for total probsets (Table S14 and S15).
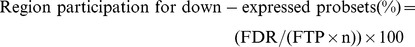
Where FDR is the frequency of region for down-expressed probsets (summation of 11 HCs), n is the number of cancers (here is 11) and FTP is the frequency of region for total probsets (Table S14 and S15). Finally, five regions with the highest ratio were selected as potential cancer-risk regions for each chromosome.

In addition, percentage of chromosome participation in differentially expressed probsets in total 11 HCs was calculated using following equations:
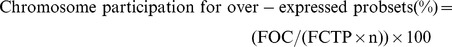
Where FOC is the frequency of chromosome for over-expressed probsets (summation of 11 HCs), n is the number of cancers (here is 11) and FCTP is the frequency of chromosome for total probsets (Table S16).
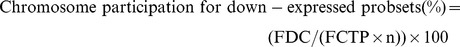
Where FDC is the frequency of chromosome for down-expressed (summation of 11 HCs), n is number of cancers (here is 11) and FCTP is the frequency of chromosome for total probsets (Table S16). Moreover, the percentages of chromosome participation for each cancer (Table S17) were calculated using fraction of chromosome frequency for altered probsets to chromosome frequency for total probsets (Table S17). The differences of chromosomes were investigated based on general chi square test.

### Construction of Networks on Common Altered MRNAs and MiRNAs

Pathway Studio 9 software (Ariadne Genomics, Rockville, MD) was used to construct different networks. Pathway Studio uses the RESNET Mammal database, which is a comprehensive pathway and molecular interaction database [46]. This database includes new aliases for human genes, miRNAs and entries from other mammals. The shortest path algorithm was used to construct four different networks based on altered mRNAs and miRNAs [47]. Five networks were constructed based on common altered RNAs, including network of commonly altered mRNAs, network of commonly altered mRNAs on PCSRs, network of commonly altered miRNAs, network of commonly altered miRNAs on PCSRs and integrative network of common altered mRNAs and miRNAs. The biological process of each network was identified using the DAVID (http://david.abcc.ncifcrf.gov/tools.jsp) suite of bioinformatics tools. DAVID bioinformatics resources consists of an integrated biological knowledgebase and analytic tools aimed at systematically extracting biological meaning from large gene/protein lists [48].

### Promoter Analysis of Altered RNAs

Promoter analysis was conducted for co-expressed mRNAs across different cancers using pscan[49]. Transcription factors (TFs) were predicted in the promoter regions (−1 kb to 0) of mRNAs using Jaspar database (TFs with P-value<0.1 were selected). In the case of miRNAs, common regulators were predicted for altered miRNAs at same region using Jaspar web tool (http://jaspar.genereg.net/). TFs were predicted in the putative promoter regions (−3 kb to +1 kb) of microRNAs with at least 99% relative profile score threshold. Expression of predicted TFs was determined using transcript-microarray expression data of 11 different cancers including breast, colorectal, endometrial, gastric, liver, lung, ovarian, pancreatic, prostate, testicular, bladder, intestine neuroendocrine, cervical and renal cancers as well as glioblastoma.

## Supporting Information

Figure S1 **Percentage of chromosome participation in gene expression.**
(PDF)

Figure S2 **Network of common altered mRNAs in variety of cancers.**
(PDF)

Figure S3 **Network of common altered mRNAs on predicted risk regions in variety of cancers.**
(PDF)

Figure S4 **Network of common altered miRNAs in variety of cancers.**
(PDF)

Figure S5 **Network of common altered miRNAs on predicted risk regions in variety of cancers.**
(PDF)

Figure S6 **Network of common altered mRNAs and microRNAs in variety of cancers.**
(PDF)

Table S1 **Number of total and differentially expressed data in different 15 cancers.**
(PDF)

Table S2 **Predicted potential cancer-susceptibility regions (PSCRs) using microarray datasets of 11 cancers.**
(PDF)

Table S3 **The percentage of chromosome participation for differentially expressed genes obtained from microarray analysis of 11 cancers.**
(PDF)

Table S4 **Properties of different constructed networks of common altered mRNAs and miRNAs in 11 cancers.**
(PDF)

Table S5 **Common entities observed between different constructed cancer networks.**
(PDF)

Table S6 **Expression patterns of common predicted regulators of over-expressed RNAs in 11 different cancers.**
(PDF)

Table S7 **Expression patterns of common predicted regulators for down-expressed RNAs in 11 different cancers.**
(PDF)

Table S8 **List of transcription factors (TFs) which were predicted in the putative promoter regions (−3 kb to +1 kb) of altered microRNAs using JASPAR.**
(PDF)

Table S9 **Expression patterns of common predicted regulators for altered microRNAs during different 11 cancers.**
(PDF)

Table S10 **Details of raw CEL (microarray chip) expression data (obtained from GEO database) have been used in present study.**
(PDF)

Table S11 **Details of EST libraries used for DDD and EST-SSR analysis (Each Library ID cell has hyperlink to NCBI Unigene library).**
(XLSX)

Table S12 **Frequency of altered probset for each chromosomal region (for probsets with at least 2 fold change) in each cancer.**
(XLSX)

Table S13 **Frequency of altered probset for each chromosomal region (first 200 probsets with highest fold changes) in each cancer.**
(XLSX)

Table S14 **Percentage of region participation for total differentially expressed probsets (with at least 2 fold changes) during total 11 cancers.**
(XLSX)

Table S15 **Percentage of region participation for total differentially expressed probsets (first 200 probsets with highest fold changes) during total 11 Cancers.**
(XLSX)

Table S16 **Percentage of chromosome participation for total differentially expressed probsets (with at least 2 fold change) during total 11 cancers.**
(XLSX)

Table S17 **Frequency of chromosome participation in gene.**
(XLSX)

Script S1 **An in-house developed python script for counting altered probsets in each chromosomal region.**
(TXT)
