# Supplementary material for: Prediction of Potential Cancer-Risk Regions Based on Transcriptome Data: Towards a Comprehensive View
Source: PLoS One. 2014 May 5;9(5):e96320. doi: 10.1371/journal.pone.0096320 (PMC4010480; doi:10.1371/journal.pone.0096320)
Supplement: Figure S1 — Percentage of chromosome participation in gene expression. (PDF) [file pone.0096320.s001.pdf]

Supplementary Figuers

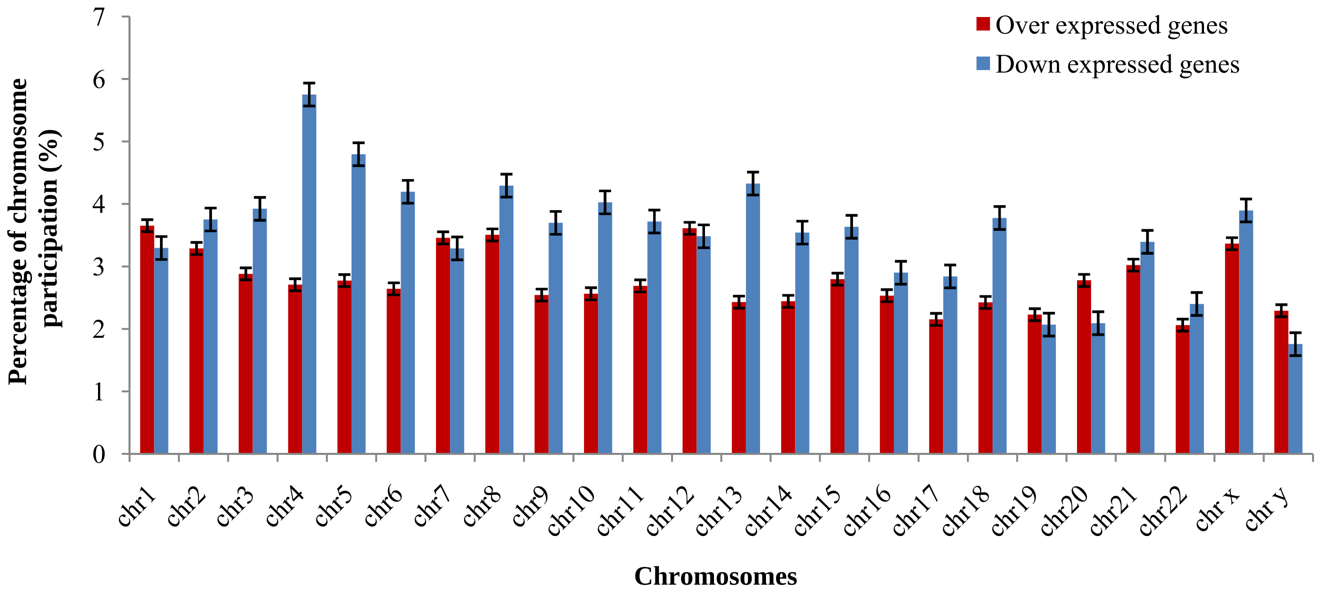

Figure S1 percentage of chromosome participation in gene expression. these percentages are summation of 11 cancers including breast, endometrial, ovarian, prostate, testicular, colorectal, gastric, liver, pancreatic, lung cancers and glioblastoma.a summary of chromosomal participation of 11 hcs shows significant differences as indicated by general chi-squared test. four top chromosomes harboring the most down-expressed genes were chrs 4, 5, 13 and x, whereas in the case of over-expressed genes the highest numbers of alteration were recorded for chrs 1, 7, 8 and 12 .
