# Supplementary material for: Prediction of Potential Cancer-Risk Regions Based on Transcriptome Data: Towards a Comprehensive View
Source: PLoS One. 2014 May 5;9(5):e96320. doi: 10.1371/journal.pone.0096320 (PMC4010480; doi:10.1371/journal.pone.0096320)
Supplement: Figure S2 — Network of common altered mRNAs in variety of cancers. (PDF) [file pone.0096320.s002.pdf]

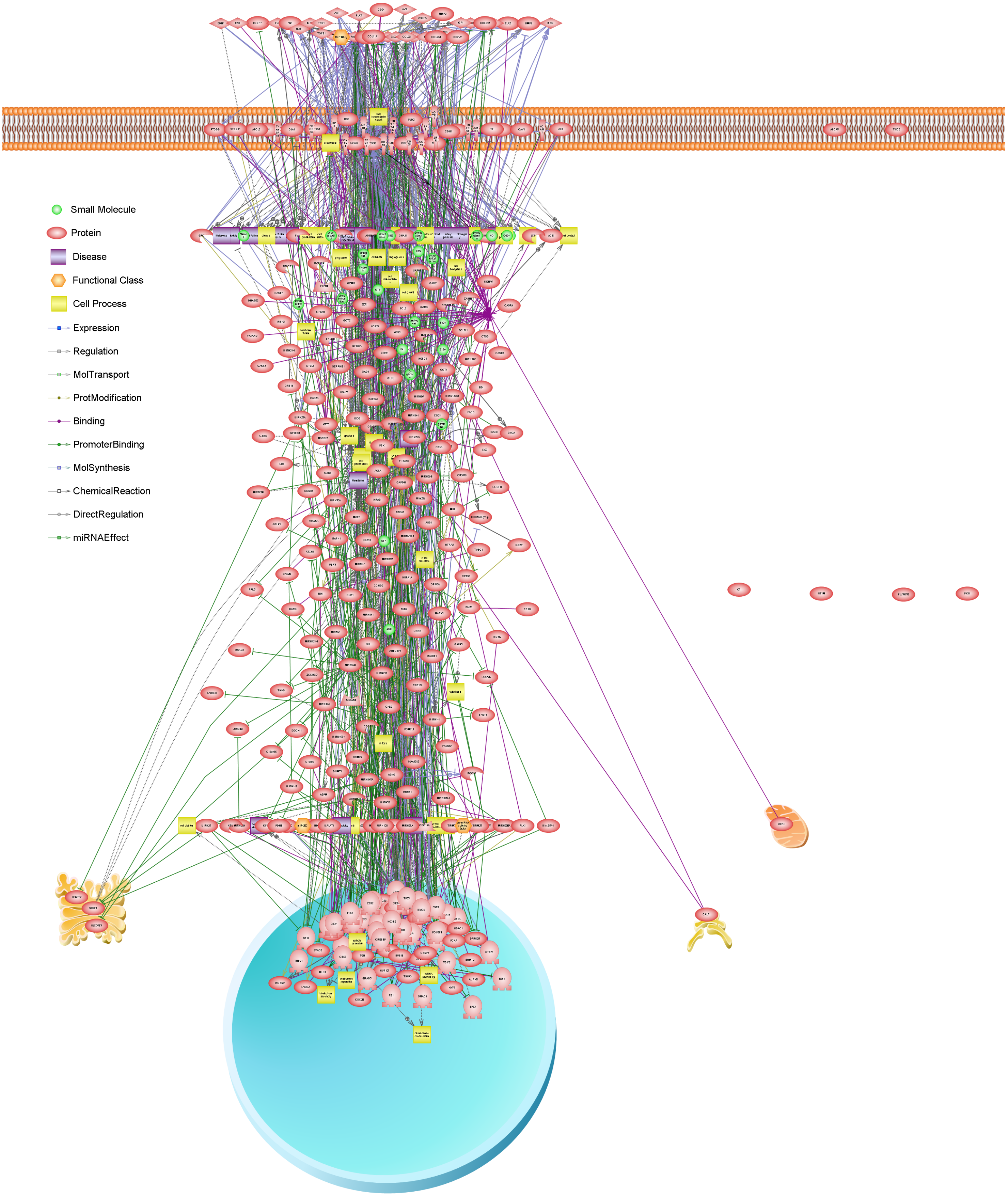

Figure S2 Network of common altered mRNAs in variety of cancers. Network was constructed using pathway studio 9 software (shortest path algorithm). This network comprises 409 entities and 1288 relations and various type of transcription factors, protein kinases, small molecules, mrnas and mirnas serve as either validated or putative regulators.
