# Supplementary material for: Prediction of Potential Cancer-Risk Regions Based on Transcriptome Data: Towards a Comprehensive View
Source: PLoS One. 2014 May 5;9(5):e96320. doi: 10.1371/journal.pone.0096320 (PMC4010480; doi:10.1371/journal.pone.0096320)
Supplement: Figure S4 — Network of common altered miRNAs in variety of cancers. (PDF) [file pone.0096320.s004.pdf]

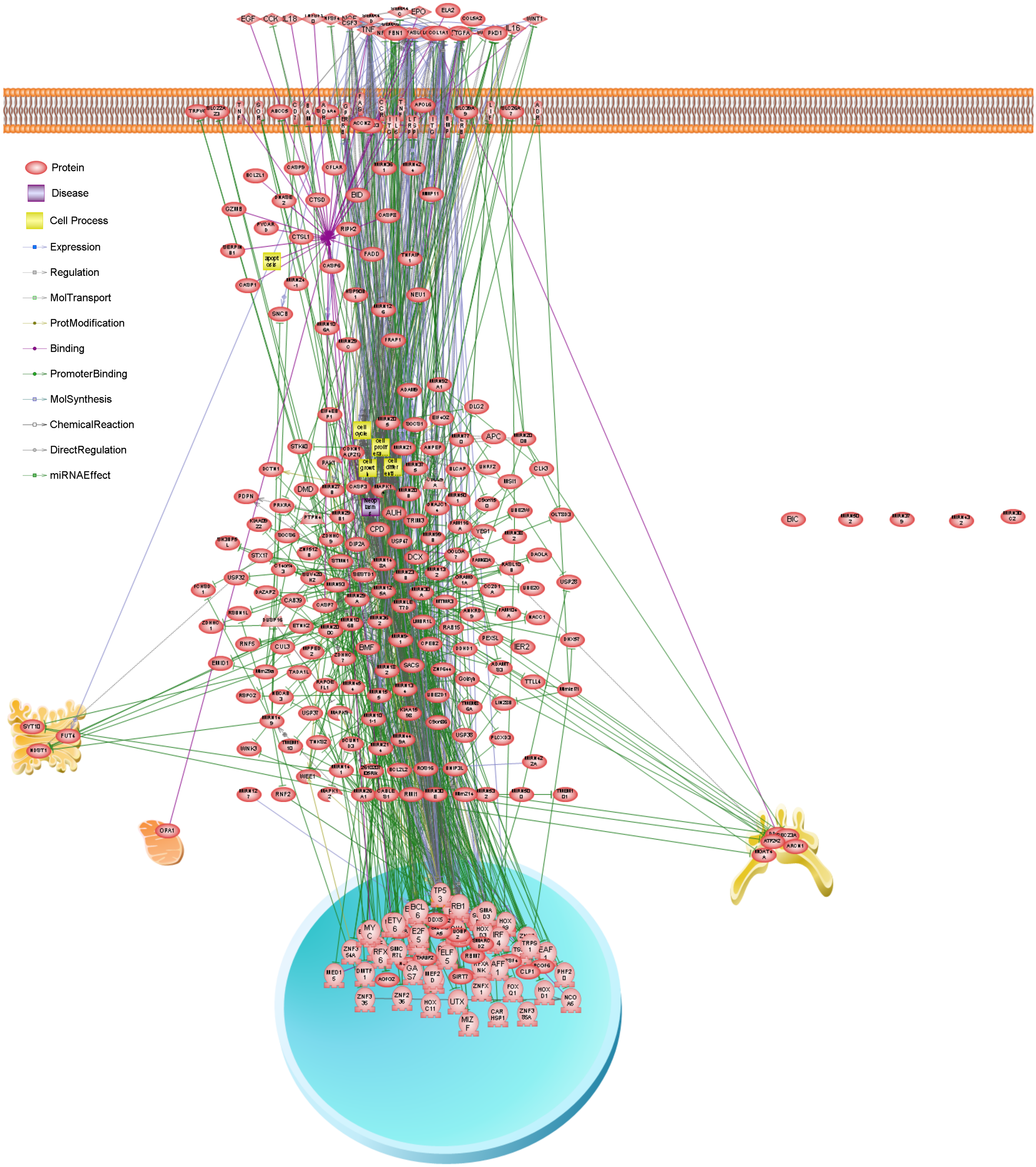

Figure S4 Network of common altered microRNAs in variety of cancers. Network was constructed using pathway studio 9 software (shortest path algorithm). This network comprises 322 entities and 1041 relations and various type of transcription factors , protein kinases , small molecules , mrnas and mirnas serve as either validated or putative regulators.
