# Supplementary material for: Prediction of Potential Cancer-Risk Regions Based on Transcriptome Data: Towards a Comprehensive View
Source: PLoS One. 2014 May 5;9(5):e96320. doi: 10.1371/journal.pone.0096320 (PMC4010480; doi:10.1371/journal.pone.0096320)
Supplement: Figure S6 — Network of common altered mRNAs and microRNAs in variety of cancers. (PDF) [file pone.0096320.s006.pdf]

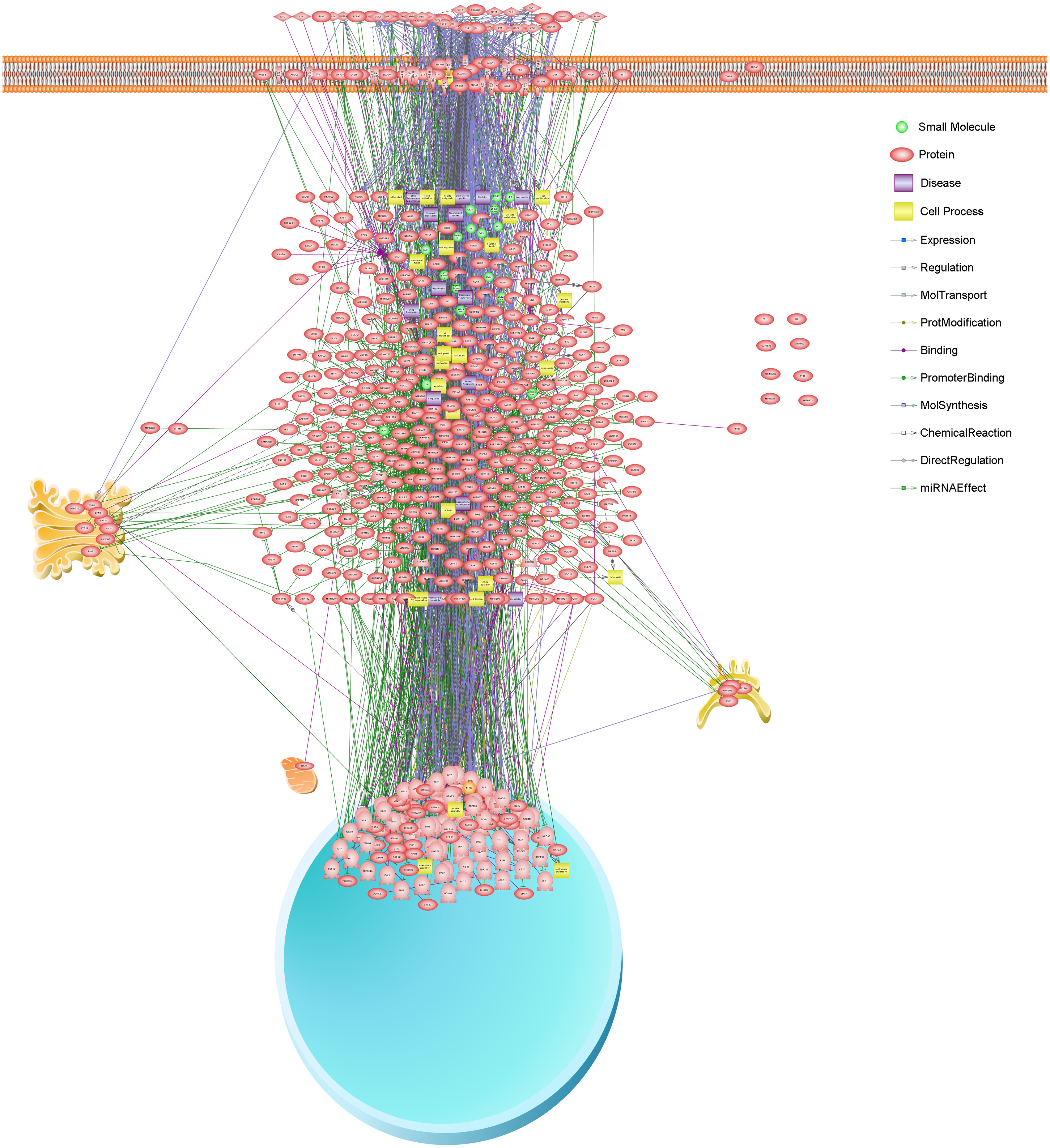

Figure S6 Network of common altered mRNAs and microRNAs in variety of cancers. Network was constructed using pathway studio7 software (shortest path algorithm). This network comprises 667 entities and 2482 relations and various type of transcription factors, protein kinases, small molecules, mrnas and mirnas serve as either validated or putative regulators.
