# Supplementary material for: Prediction of Potential Cancer-Risk Regions Based on Transcriptome Data: Towards a Comprehensive View
Source: PLoS One. 2014 May 5;9(5):e96320. doi: 10.1371/journal.pone.0096320 (PMC4010480; doi:10.1371/journal.pone.0096320)
Supplement: Table S1 — Number of total and differentially expressed data in different 15 cancers. (PDF) [file pone.0096320.s007.pdf]

Table S1 Number of total and differentially expressed data in different cancers. The number of over-expressed transcripts were ranged from minimum 6 in colorectal and renal cancers (EST data and miRNA expression, respectively) to maximum 6743 in testicular cancer (transcript microarray datasets). In contrast, the range of down-expressed transcripts changed from 4 in ovarian cancer to 4984 in testicular cancer (EST data and transcript microarray datasets, respectively).

	Number of total and differentially expressed Data									
	Transcript microarray datasets			Digital differential display of EST libraries				MicroRNA expression datasets		
Cancer	Total Probsets	OE[a] Probsets (2FC[c])	DE[b] Probsets (2FC)	Total ESTs[d]	Total Unigenes	OE Unigenes	DE Unigenes	Total Probsets	OE Probsets (1.5 FC)	DE Probsets (1.5 FC)
Breast	54675	1283	1672	44433	130	77	53	-	-	-
Endometrial	54675	1113	2024	103974	251	136	115	-	-	-
Ovarian	54675	1219	4865	18335	12	8	4	574	71	22
Prostate	54675	557	582	84272	189	129	60	830	19	16
Testicular	54675	6743	4984	106842	307	165	142	-	-	-
Colorectal	54675	530	1490	37467	71	6	65	-	-	-
Gastric	54675	528	547	28373	88	41	47	-	-	-
Liver	54675	1365	1283	87656	238	148	90	-	-	-
Pancreatic	54675	274	159	145115	457	301	156	-	-	-
Brain	54675	1560	1133	128031	467	201	266	-	-	-
Lung	54675	1974	2663	137465	236	125	111	-	-	-
Renal	-	-	-	-	-	-	-	830	6	72
Cervical	-	-	-	-	-	-	-	1082	50	136
Bladder	-	-	-	-	-	-	-	830	33	10
intestine neuroendocrine tumors	-	-	-	-	-	-	-	830	67	59

[a]Over expressed. [b]Down expressed. [c]Fold change. [d]Summation of both normal and cancerous EST libraries.
