# Supplementary material for: Prediction of Potential Cancer-Risk Regions Based on Transcriptome Data: Towards a Comprehensive View
Source: PLoS One. 2014 May 5;9(5):e96320. doi: 10.1371/journal.pone.0096320 (PMC4010480; doi:10.1371/journal.pone.0096320)
Supplement: Table S2 — Predicted potential cancer-susceptibility regions (PSCRs) using microarray datasets of 11 cancers. (PDF) [file pone.0096320.s008.pdf]

Table S2 Predicted potential cancer-susceptibility regions (PSCRs) using microarray datasets of 11 cancers including, breast, endometrial, ovarian, prostate, testicular, colorectal, liver, gastric, pancreatic, lung cancers and glioblastoma . The percentage of region participation in cancer was calculated for first 200 probset with higher symmetrical fold changes.

Chromosome		Five Top Regions with highest percentage (%)									
		**Over-expressed**					**Down-expressed**				
**1**	Region	p11.2	p21.1	p31.2	q31.3	q32.3	p21.1	p22.3	q24.3	q31.1	q31.3
	Percentage	3.79	6.22	9.09	2.22	1.41	0.96	1.12	1.06	2.16	1.11
**2**	Region	p24.2	p25.2	q12.3	q22.2	q32.2	p12	p23.1	p25.2	q12.2	q32.3
	Percentage	1.21	2.27	1.62	1.52	1.44	1.65	1.65	1.36	1.21	1.14
**3**	Region	q12.1	q24	q25.33	q26.2	q28	p12.1	p12.3	q12.2	q25.2	q26.1
	Percentage	1.89	1.16	1.25	0.86	1.65	1.82	1.65	3.64	1.58	2.27
**4**	Region	p15.1	p15.31	q22.3	q28.3	q32.1	q13.3	q23	q27	q34.1	q34.2
	Percentage	3.64	1.58	2.8	1.3	1.14	2.47	2.62	2.05	3.39	7.58
**5**	Region	q12.1	q13.3	q14.2	q31.2	q33.2	p13.1	p14.2	p15.1	q22.1	q22.3
	Percentage	0.97	1.49	1.52	1.07	0.93	3.03	1.82	0.85	1.14	0.79
**6**	Region	p21.32	p25.3	q13	q22.1	q22.32	p24.1	q14.2	q14.3	q16.1	q22.31
	Percentage	1.54	1.14	1.44	1.82	0.83	1.73	2.42	1.21	1.76	1.92
**7**	Region	p14.1	p14.2	p15.2	p15.3	p21.1	p15.1	p21.2	q21.11	q21.3	q31.2
	Percentage	2.83	2.14	1.44	1.26	1.69	1.82	1.14	2.6	1.65	2.05
**8**	Region	q13.2	q21.13	q22.1	q22.3	q24.22	p21.1	p22	q21.11	q21.2	q23.2
	Percentage	4.22	2.18	1.11	1.12	1.22	2.07	1.34	1.68	1.82	3.64
**9**	Region	q21.2	q22.2	q31.2	q33.1	q34.12	q21.11	q21.12	q21.13	q22.31	q33.1
	Percentage	1.52	1.82	1.38	0.87	1.21	1.27	1.7	0.92	1.32	2.81
**10**	Region	p15.2	q21.1	q21.2	q23.33	q26.2	q21.1	q22.3	q23.2	q23.33	q25.3
	Percentage	1.01	1.27	2.56	2.16	1.95	1.27	0.88	0.81	1.15	1.03
**11**	Region	p11.12	p14.2	p15.2	q14.3	q22.2	p14.1	p14.2	p15.3	q23.2	q24.1
	Percentage	1.82	1.4	0.83	0.91	7.58	1.19	1.4	1.11	2.31	1.04
**12**	Region	p11.22	p12.2	p13.31	q13.11	q21.1	p12.3	p13.1	q14.3	q21.1	q21.33
	Region	1.1	1.95	0.82	0.8	0.88	1.12	0.95	1.07	1.17	2.39
**13**	Percentage	q13.2	q13.3	q21.1	q22.3	q31.2	q13.3	q14.11	q21.1	q21.32	q22.3
	Region	2.27	1.15	1.14	1.14	1.3	2.02	0.51	2.27	1.3	2.65
**14**	Percentage	q11.2	q22.1	q22.2	q22.3	q23.1	q23.2	q24.1	q31.1	q31.3	q32.2
	Region	0.85	0.6	1.82	0.6	0.9	0.73	0.51	0.78	0.95	0.84
**15**	Percentage	q12	q13.3	q15.1	q21.3	q22.2	q12	q13.3	q21.1	q22.33	q26.2
	Region	1.82	1.86	1.01	1.4	1.02	0.91	0.93	0.98	1.14	1.82
**16**	Percentage	p12.3	q12.1	q12.2	q21	q24.2	p12.3	p13.11	q12.2	q13	q23.2
	Region	0.62	0.68	0.46	1.03	0.49	1.14	0.86	1.5	0.97	0.68
**17**	Percentage	p12	q21.2	q21.33	q22	q24.3	p11.2	q21.33	q23.1	q24.2	q24.3
	Region	0.48	1.08	0.64	0.54	1.75	0.38	0.32	1.17	1.3	0.7
**18**	Percentage	p11.22	p11.32	q12.1	q21.31	q21.33	p11.31	q11.2	q12.1	q21.2	q21.31
	Region	0.71	0.89	0.97	0.7	1.22	1.1	1.65	0.73	0.61	0.7
**19**	Percentage	p12	q13.2	q13.32	q13.33	q13.41	p13.2	p13.3	q12	q13.2	q13.32
	Region	0.57	0.41	0.45	0.36	0.38	0.26	0.18	0.88	0.66	0.25
**20**	Percentage	p11.21	p12.3	q11.23	q13.2	q13.31	p11.21	p12.1	p12.3	q11.23	q13.31
	Region	0.92	0.7	0.71	2.14	2.05	0.26	0.44	0.56	0.24	0.59
**21**	Percentage	p11.1	p11.2	q11.2	q22.12	q22.3	q21.1	q21.3	q22.11	q22.12	q22.13
	Region	1.52	1.01	0.53	0.87	0.45	0.19	1.01	0.21	0.43	1.27
**22**	Percentage	q11.23	q12.1	q12.2	q12.3	q13.32	q11.22	q11.23	q12.1	q13.1	q13.33
	Region	0.2	0.25	0.12	0.08	1.52	0.46	0.1	0.49	0.25	0.22
**x**	Percentage	p21.1	p22.32	q12	q22.1	q26.2	p22.32	q21.32	q22.1	q22.3	q23
	Region	1.52	2.27	1.24	0.88	3.31	2.27	6.06	1.64	2.27	1.88
**y**	Region	p11.31	q11.222				p11.31	q11.223	q11.23		
	Percentage	0.7	2.89				0.7	1.65	2.27		

[a]Percentage: the fraction of altered probsets frequency for each region to the correspondence frequency of total probsets on microarray chip at the same region.
