# Supplementary material for: Prediction of Potential Cancer-Risk Regions Based on Transcriptome Data: Towards a Comprehensive View
Source: PLoS One. 2014 May 5;9(5):e96320. doi: 10.1371/journal.pone.0096320 (PMC4010480; doi:10.1371/journal.pone.0096320)
Supplement: Table S3 — The percentage of chromosome participation for differentially expressed genes obtained from microarray analysis of 11 cancers. (PDF) [file pone.0096320.s009.pdf]

Table S3 The percentage of chromosome participation for differentially expressed genes obtained from microarray analysis of 11 cancers including breast, endometrial, ovarian, prostate, testicular, colorectal, liver, gastric, pancreatic, lung cancers and glioblastoma. chr4 is harboring the highest number of genes altered in cancer (excluding prostate and gastric cancers). In contrast, chrY has the lowest number of genes expressed in cancer.

Chr.	Breast		Endometrial		Ovarian		Prostate		Testicular		Brain		Lung		Colorectal		Gastric		Liver		Pancreatic	
	[a]DE	[b]OE	DE	OE	DE	OE	DE	OE	DE	OE	DE	OE	DE	OE	DE	OE	DE	OE	DE	OE	DE	OE
1	[c]2.82	2.41	3.11	2.74	8.65	2.65	1.10	0.78	7.39	17.64	1.77	2.97	4.81	4.01	2.70	0.97	1.31	1.04	2.24	4.40	0.35	0.58
2	3.53	2.67	3.84	3.03	8.83	2.64	1.20	1.07	9.93	15.42	2.25	2.59	4.97	3.89	2.98	0.71	1.20	1.41	2.17	2.14	0.37	0.60
3	3.56	1.83	3.90	1.29	9.29	2.64	1.57	0.85	10.45	11.77	2.39	3.40	5.57	5.38	3.02	0.60	0.82	0.82	2.36	2.64	0.22	0.47
4	4.24	2.75	5.59	2.07	14.96	1.85	1.35	1.53	14.02	10.59	3.38	3.06	6.80	2.97	5.63	1.13	1.67	1.04	5.05	1.71	0.59	1.08
5	4.00	3.29	4.47	2.82	11.99	2.27	1.14	1.25	15.24	9.48	2.70	2.43	5.84	3.41	3.80	1.02	1.29	1.29	2.27	3.02	0.04	0.24
6	3.62	2.69	3.87	2.14	9.77	1.76	1.24	1.17	12.08	6.77	2.52	4.11	6.63	3.87	3.04	1.04	0.97	0.86	2.17	4.07	0.24	0.59
7	3.66	2.15	3.63	1.22	8.58	2.03	1.15	1.33	6.70	15.32	1.55	5.51	4.59	3.33	2.52	1.41	0.96	1.70	2.40	3.18	0.44	0.85
8	4.67	2.59	4.87	2.59	9.79	3.86	1.17	1.62	13.04	10.71	2.99	3.81	5.02	5.23	2.49	1.73	0.71	1.37	2.33	4.57	0.15	0.51
9	3.25	2.35	4.24	1.51	9.98	1.41	0.94	0.89	8.15	13.47	2.07	2.59	4.94	2.64	2.54	0.61	0.89	0.47	3.39	1.32	0.28	0.71
10	3.22	2.72	3.67	2.49	8.25	2.09	0.86	0.77	12.38	8.70	3.76	1.99	5.26	3.81	2.99	0.82	1.27	0.95	2.36	3.31	0.27	0.54
11	2.89	2.59	3.37	2.01	8.27	3.00	1.19	1.36	10.89	9.39	1.67	3.34	5.55	3.37	2.79	0.88	1.09	0.99	2.93	2.14	0.27	0.51
12	2.99	2.41	3.85	2.38	8.64	2.45	1.15	1.15	7.06	19.45	2.70	2.95	4.83	4.50	2.77	0.94	0.76	0.72	3.31	2.16	0.25	0.61
13	3.76	2.16	5.76	2.00	12.00	1.84	1.04	2.16	10.80	9.60	2.80	2.16	6.56	1.52	1.76	2.08	0.32	1.12	2.72	1.52	0.08	0.56
14	3.40	1.78	2.79	2.45	9.48	2.23	0.78	0.78	9.70	8.42	1.73	1.73	3.68	4.29	3.96	0.61	1.23	1.17	1.67	3.07	0.56	0.33
15	2.43	2.60	4.19	2.26	11.26	2.09	1.02	1.02	9.05	12.67	2.71	1.70	4.07	3.51	2.71	0.57	0.85	1.02	1.41	2.88	0.28	0.45
16	1.69	2.80	2.89	1.54	7.37	1.54	0.77	0.82	5.01	14.75	1.30	1.59	4.34	2.22	2.75	0.72	1.54	0.43	3.47	1.06	0.77	0.39
17	2.12	1.84	2.70	1.73	7.25	1.70	0.94	0.52	8.77	8.11	1.25	2.70	4.09	3.50	1.60	0.97	0.73	0.94	1.60	1.42	0.21	0.24
18	3.08	2.05	3.18	1.33	12.51	1.13	1.33	0.82	7.59	8.82	2.56	2.87	4.21	5.74	4.10	0.51	1.13	1.03	1.44	2.05	0.41	0.31
19	1.30	1.89	3.29	1.16	3.85	2.07	0.49	0.53	5.81	12.89	0.60	1.65	3.85	1.96	1.02	0.77	0.49	0.56	2.00	0.67	0.07	0.39
20	2.84	1.72	2.58	2.12	5.29	2.71	0.66	0.53	4.37	10.65	1.19	3.04	4.03	3.37	0.73	2.91	0.26	1.26	1.06	1.92	0.00	0.33
21	3.07	2.78	5.71	1.61	7.03	3.51	0.73	2.20	7.91	15.52	1.17	2.34	4.69	1.46	2.64	1.02	2.05	0.59	2.20	1.61	0.15	0.59
22	2.99	2.58	1.86	1.37	4.52	1.37	0.40	0.48	8.23	8.31	0.89	1.69	2.42	4.68	2.82	0.40	1.05	0.48	0.89	1.29	0.32	0.00
X	3.88	2.15	5.31	1.67	11.28	1.79	1.85	0.66	7.10	18.39	2.15	3.82	5.19	4.06	3.10	1.13	0.60	0.78	1.97	2.15	0.42	0.42
Y	0.00	0.00	0.00	0.00	2.52	2.52	0.84	0.84	7.56	8.40	0.00	8.40	3.36	2.52	0.00	0.00	5.04	0.00	0.00	2.52	0.00	0.00

[a]Down expressed. [b]Over expressed. [C] Percentage of chromosome participation
