# Supplementary material for: Prediction of Potential Cancer-Risk Regions Based on Transcriptome Data: Towards a Comprehensive View
Source: PLoS One. 2014 May 5;9(5):e96320. doi: 10.1371/journal.pone.0096320 (PMC4010480; doi:10.1371/journal.pone.0096320)
Supplement: Table S4 — Properties of different constructed networks of common altered mRNAs and miRNAs in 11 cancers. (PDF) [file pone.0096320.s010.pdf]

Table S4 Properties of different constructed networks of common altered mRNAs and miRNAs in 11 cancers(breast, colorectal, endometrial, gastric, liver, lung, ovarian, pancreatic, prostate and testicular cancers as well as glioblastoma). All networks were constructed using pathway studio 9 software (shortest path algorithm). Networks have similar biological processes, such as cellular process, biological regulation, metabolic process, multicellular organismal process, developmental process and response to stimulus.

Network	Number of imported variants	Total Number of entity in network	Total Number of relations in network	[c] Biological processes of network
**[a]Common altered genes**	42	409	1288	cellular process, biological regulation, metabolic process, multicellular organismal process, developmental process, response to stimulus, cellular component organization, localization, immune system process, death, reproductive process, reproduction, cellular component, biogenesis, multi-organism process, locomotion, biological adhesion, growth, rhythmic process, pigmentation
**[a]Common altered genes on predicted cancer-risk loci**	31	383	1121	cellular process, biological regulation, metabolic process, multicellular organismal process, developmental process, response to stimulus cellular component organization, localization, establishment of localization, immune system process, death, reproductive process, reproduction, multi-organism process, biological adhesion, locomotion, cellular component biogenesis, growth, rhythmic process, pigmentation
**[b]Common altered microRNAs**	40	322	1041	cellular process, biological regulation, metabolic process, multicellular organismal process, developmental process, response to stimulus, cellular component organization, localization, establishment of localization, immune system process, death, reproductive process, reproduction, multi-organism process, biological adhesion, locomotion, cellular component biogenesis, growth, rhythmic process, pigmentation
**[b]Common altered microRNAs on predicted cancer-risk loci**	15	123	409	cellular process, biological regulation, metabolic process, multicellular organismal process, developmental process, response to stimulus, cellular component organization, immune system process, locomotion, multi-organism process
**Total altered mRNAs and microRNAs**	82	667	2482	cellular process, biological regulation, metabolic process, multicellular organismal process, developmental process, cellular component organization, response to stimulus, localization, immune system process, death, cellular component biogenesis, multi-organism process reproduction, reproductive process, locomotion, growth, rhythmic process

[a] See Table 1; [b] See Table 2; [c] From the highest count to the lowest
