# Supplementary material for: Prediction of Potential Cancer-Risk Regions Based on Transcriptome Data: Towards a Comprehensive View
Source: PLoS One. 2014 May 5;9(5):e96320. doi: 10.1371/journal.pone.0096320 (PMC4010480; doi:10.1371/journal.pone.0096320)
Supplement: Table S5 — Common entities observed between different constructed cancer networks. (PDF) [file pone.0096320.s011.pdf]

Table S5 Common entities observed between different constructed cancer networks. Zinc finger E-box binding homeobox 2 (ZEB2), DEAD (Asp-Glu-Ala-Asp) box helicase 5 (DDX5) and leukemia inhibitory factor receptor alpha (LIFR) and also mir-21, mir-30a, mir-141 and mir-200c were shared between both constructed networks of common altered mRNAs and miRNAs (from 11 cancers: breast, colorectal, endometrial, gastric, liver, lung, ovarian, pancreatic, prostate and testicular cancers as well as glioblastoma).

**Common entities between network of total genes and network of total microRNAs**			**Common entities between network of genes on predicted cancer risk loci and network of total microRNAs**			**Common entities between network of genes on predicted cancer risk loci and network of microRNAs on predicted cancer risk loci**	**Common entities between network of genes and network of microRNAs on predicted cancer risk loci**
ADRB2	E2F1	MIRN29B1	ADRB2	ELA2	MIRN30A	ADRB2	ADRB2
APOL6	EGF	MIRN29C	APOL6	EPO	MIRN9-1	ATXN1	ATXN1
apoptosis	ELA2	MIRN30A	apoptosis	FADD	MYC	cell differentiation	cell differentiation
BCL2L1	EPO	MIRN9-1	BCL2L1	FAS	Neoplasms	cell growth	cell growth
BID	FADD	MYC	BID	FASLG	NF-kB	cell proliferation	cell proliferation
CASP1	FAS	Neoplasms	CASP1	FBN1	OPA1	CRKL	CRKL
CASP3	FASLG	NF-kB	CASP3	FN1	PYCARD	FN1	DDX5
CASP6	FN1	NGF	CASP6	GZMB	RIPK2	MIRN141	FN1
CASP7	GZMB	OPA1	CASP7	HOXA5	TGFB1	MIRN200C	MAPK14
CASP8	HOXA5	PYCARD	CASP8	IL18	TNF	MIRN21	MIRN141
CASP9	IL18	RB1	CASP9	LIFR	TNFRSF10B	MIRN30A	MIRN200C
CCR5	LIFR	RIPK2	CCR5	MIRN141	TNFRSF1A	Neoplasms	MIRN21
cell cycle	MAPK14	SERPINB1	CDKN1A (P21)	MIRN148A	TNFSF10	NF-kB	MIRN30A
cell differentiation	MIRN141	SMAD3	cell cycle	MIRN182	TP53	TGFB1	Neoplasms
cell growth	MIRN148A	TGFB1	cell differentiation	Mirn200b	TRPS1	TNF	NF-kB
cell proliferation	MIRN182	TNF	cell growth	MIRN200C	VEGFA	TRPS1	RB1
CFLAR	MIRN200B	TNFRSF10B	cell proliferation	MIRN21	YES1	VEGFA	SMAD3
COL1A1	MIRN200C	TNFRSF1A	CFLAR	MIRN24-1		ZEB2	TGFB1
CTSD	MIRN21	TNFSF10	CTSD	MIRN27B			TNF
CTSL1	MIRN24-1	TP53	CTSL1	Mirn29a			TRPS1
DDX5	MIRN27B	TRPS1	DNASE2	MIRN29B1			VEGFA
DNASE2	Mirn29a	VEGFA	EGF	MIRN29C			ZEB2
