# Supplementary material for: Prediction of Potential Cancer-Risk Regions Based on Transcriptome Data: Towards a Comprehensive View
Source: PLoS One. 2014 May 5;9(5):e96320. doi: 10.1371/journal.pone.0096320 (PMC4010480; doi:10.1371/journal.pone.0096320)
Supplement: Table S6 — Expression patterns of common predicted regulators of over-expressed RNAs in 11 different cancers. (PDF) [file pone.0096320.s012.pdf]

Table S6 Expression patterns of common predicted regulators of over-expressed RNAs in different cancers (cancer data of 11 microarray studies: breast, colorectal, endometrial, gastric, liver, lung, ovarian, pancreatic, prostate and testicular cancers as well as glioblastoma). Among 18 common predicted TFs for over-expressed mRNAs, Kruppel-Like Factor 4 (KLF4) located at PCSRs was found to be down-expressed in 7 types of cancers.

Gene symbol	Region	Pvalue	[a]PCSR	Breast	Endometrial	Ovarian	Prostate	Testicular	Colorectal	Liver	Gastric	Pancreatic	Glioblastoma	Lung
**NFYA**	6p21.1	0.00004	-	↓	↑	↑		↑		↑	↑	↓		↑
**INSM1**	20p11.23	0.02443	-						↓					↑
**Gfi1**	1p22.1	0.02938	-			↑		↓	↑					
**AP1 (JUN)**	chr1p32.1	0.04677	-	↓	↓	↑		↓	↓					↓
**Arnt**	1q21.3	0.04896	-			↓		↓	↓	↑				↓
**Ahr**	7p21.1	0.04896	-		↓		↑	↓			↑		↓	↓
**Pax4**	7q32.1	0.05596	-					↓						
**Nr2e3**	15q23	0.06206	-		↑						↓			
**MEF2A**	15q26.3	0.06565	-		↓	↓		↓	↓		↑		↑	↓
**PPARG**	3p25.2	0.07117	-	↓			↓			↑		↑		↓
**RXRA**	9q34.2	0.07117	-			↓		↑	↑	↓			↑	↓
**HNF4A**	20q13.12	0.07563	-			↑		↓		↓	↑		↓	↓
**NFE2L1**	17q21.32	0.08202	-		↑	↓		↓	↓					↑
**MafG**	17q25.3	0.08202	-		↑			↓	↑					↑
**Pdx1**	13q12.2	0.08274	-			↑		↓			↓			
**HNF1A**	12q24.31	0.08293	-						↑					
**Klf4**	9q31.2	0.08677	✓	↓	↓	↓			↓	↓	↓			↓
**Prrx2**	9q34.11	0.08683	-			↑		↑						↑

[a] Predicted cancer- susceptibility region
