# Supplementary material for: Prediction of Potential Cancer-Risk Regions Based on Transcriptome Data: Towards a Comprehensive View
Source: PLoS One. 2014 May 5;9(5):e96320. doi: 10.1371/journal.pone.0096320 (PMC4010480; doi:10.1371/journal.pone.0096320)
Supplement: Table S7 — Expression patterns of common predicted regulators for down-expressed RNAs in 11 different cancers. (PDF) [file pone.0096320.s013.pdf]

Table S7 Expression patterns of common predicted regulators for down-expressed RNAs in different cancers (cancer data of 11 microarray studies: breast, colorectal, endometrial, gastric, liver, lung, ovarian, pancreatic, prostate and testicular cancers as well as glioblastoma). From total 13 common regulators predicted for down-expressed mRNAs, 6 regulators are located on PCSRs.

Gene symbol	Region	Pvalue	[a]PCSR	Breast	Endometrial	Ovarian	Prostate	Testicular	Colorectal	Liver	Gastric	Pancreatic	Glioblastoma	Lung
**FOXD1**	5q13.2	0.0008	✓			↑	↑	↓	↑		↑			↑
**PBX1**	1q23.3	0.0204	-	↓	↓	↓		↑	↓		↓			↓
**NFIL3**	9q22.31	0.0211	✓	↓		↓			↓		↑			↓
**TBP**	6q27	0.0307	-		↓	↓		↑						
**Gfi1**	1p22.1	0.0419	-			↑		↓	↑					
**FOXA1**	14q21.1	0.0431	✓	↑	↑	↑	↑	↓	↓		↓	↑		↑
**CEBPA**	19q13.11	0.0595	-	↑				↑						↓
**FOXA2**	20p11.21	0.0703	✓		↓	↑		↓	↑		↓			↓
**RORA**	15q22.2	0.0850	✓	↓	↓	↓		↓	↓	↓		↓	↑	↓
**NR3C1**	5q31.3	0.0866	-		↓	↓		↓	↓					↓
**GATA2**	3q21.3	0.0903	-		↓									↓
**MEF2A**	15q26.3	0.0922	-	↓	↓		↓	↓		↑		↑	↓	↓
**TEAD1**	11p15.3	0.0995	✓			↓		↓				↑		

[a] Predicted cancer- susceptibility region
