# Supplementary material for: Prediction of Potential Cancer-Risk Regions Based on Transcriptome Data: Towards a Comprehensive View
Source: PLoS One. 2014 May 5;9(5):e96320. doi: 10.1371/journal.pone.0096320 (PMC4010480; doi:10.1371/journal.pone.0096320)
Supplement: Table S8 — List of transcription factors (TFs) which were predicted in the putative promoter regions (−3 kb to +1 kb) of altered microRNAs using JASPAR. (PDF) [file pone.0096320.s014.pdf]

Table S8 List of transcription factors (TFs) which were predicted in the putative promoter regions (-3kbto +1kb) of altered microRNAs using JASPAR (with at least 99% relative profile score threshold). Only common TFs for group of these microRNAs are presented.

MicroRNA	Cancer cell type															location	miRNA Cluster	PCSR	Common predicted TFs for miRNAs on same locus
	Br	Ce	En	Ov	Pr	Te	Re	Co	Ga	Li	Pn	Gl	Ln	In	Bl				
hsa-mir-200c		↑		↑	↑		↓								↑	12p13.31	12p13.31	✓	**NFIC, ETS1, USF1, MZF1_1-4, AP1, NR4A2,**
hsa-mir-141				↑	↑		↓								↑	12p13.31		✓	**GATA2, SOX10, FEV, SPI1, ZNF354C, SPIB**
hsa-mir-106a				↑	↑		↓								↑	Xq26.2	Xq26.2	✓	**ETS1, FOXL1, GATA2, ZNF354C, YY1, SPI1,**
hsa-mir-20b				↑	↑		↓								↑	Xq26.2		✓	**GATA3, NFATC2, AP1, NFIC, USF1**
hsa-mir-30c-2	↓	↓	↓	↓		↓	↓	↓					↓			6q13	6q13	✓	**NFIC, ETS1, MZF1_1-4, SOX10**
hsa-mir-30a		↓					↓								↓	6q13		✓	**GATA2, ZNF354C, FOXD1**
hsa-mir-500		↓					↓							↑	↑	Xp11.23		-	**ETS1 , MZF1_1-4, GATA2 ,SOX10,**
hsa-mir-532		↓					↓							↑		Xp11.23		-	**YY1 , ZNF354C , SPI1, GATA3**
hsa-mir-501		↓					↓								↑	Xp11.23	Xp11.23	-	
hsa-mir-502		↓					↓							↑		Xp11.23		-	
hsa-mir-362		↓					↓								↑	Xp11.23		-	
hsa-mir-432		↓					↓									14q32.2		✓	**YY1, ETS1, MZF1_1-4, SPIB, ZNF354C, GATA3, SOX10**
hsa-mir-770						↓		↓		↓						14q32.2	14q32.2	✓	
hsa-mir-127		↓					↓								↓	14q32.2		✓	
hsa-mir-379		↓					↓									14q32.31		-	**NFIC, ETS1, YY1, MZF1_1-4 , GATA2,**
hsa-mir-382		↓					↓								↓	14q32.31	14q32.31	-	**GATA3, ZNF354C , NFATC2, SPI1**
hsa-mir-134		↓					↓								↓	14q32.31		-	
hsa-mir-27b		↓					↓							↑		9q22.32		-	**YY1, GATA2, ETS1, NFIC, SPI1**
hsa-let-7d			↓	↓		↑	↓						↓		↑	9q22.32	9q22.32	-	**MZF1_1-4 , ZNF354C**
hsa-mir-23b		↓					↓									9q22.32		-	
hsa-mir-99b		↓			↓		↓								↑	19q13.41	19q13.41	✓	**ZNF354C, SPIB, MZF1_1-4, SP1, NR4A2, TFAP2A**
hsa-mir-125a		↓			↓		↓								↑	19q13.41		✓	**ETS1, SPI1, ELK1, NFIC, YY1**
hsa-mir-424		↓					↓									Xq26.3	-	-	**-**
hsa-mir-106b				↑	↑		↑							↑	↑	7q22.1	7q22.1	-	**MZF1_1-4 , YY1, GATA2, ZNF354C, NFATC2, HOXA5,**
hsa-mir-93				↑	↑		↑								↑	7q22.1		-	**ETS1,FOXO3, GATA3, FEV, SPI1, NFIC**
hsa-mir-149		↓					↓								↓↔↑	2q37.3	-	-	**-**
hsa-mir-200b				↑	↑		↓								↑	1p36.33	-	-	**-**
hsa-mir-21	↑			↑	↑	↓	↑		↓						↑	17q23.1	-	✓	**-**
hsa-mir-126		↑		↑												9q34.3	-	-	**-**
hsa-mir-214	↑	↑	↓			↓	↓			↓		↓			↓	1q24.3	-	✓	**-**
hsa-mir-101-1			↓	↓		↑					↑	↑	↓	↑		1p31.3	**-**	✓	**-**
hsa-miR-182				↑	↑									↑	↑	7q32.2	**-**	-	**-**
mir155HG (BIC)	↑		↓	↑		↓			↓	↑		↓	↓			21q21.3	**-**	✓	**-**
hsa-mir-30e		↓					↓							↑	↑	1p34.2	**-**	-	**-**
hsa-mir-422a		↓					↓							↓	↑	15q22.31	**-**	-	**-**
hsa-mir-132				↓			↓								↓	17p13.3	**-**	-	**-**
hsa-mir-205		↓		↓↔↑	↓										↓↔↑	1q32.2	**-**	-	**-**
hsa-mir-375		↑			↑											2q35	**-**	-	**-**
hsa-mir-1274b		↑			↑										↑	19q13.43	**-**	-	**-**
hsa-mir-361		↓					↓							↑	↓↔↑	Xq21.2	**-**	-	**-**

Abbreviations: Br, Breast; Ce, Cervical; En, Endometrial; Ov, Ovarian; Pr, Prostate; Te, Testicular; Re, Renal; Co, Colorectal; Ga, Gastric; Li, Liver; Pn, Pancreatic; Gl, Glioblastoma; Ln, Lung; In, Intestinal Neuroendocrine; Bl, Bladder; PCSR, Potential Cancer-Susceptibility Region; TF, Transcription Factor.
Symbols: ↑, over-expression; ↓, down-expression; ✓, risk region
