# Supplementary material for: Prediction of Potential Cancer-Risk Regions Based on Transcriptome Data: Towards a Comprehensive View
Source: PLoS One. 2014 May 5;9(5):e96320. doi: 10.1371/journal.pone.0096320 (PMC4010480; doi:10.1371/journal.pone.0096320)
Supplement: Figure S5 — Network of common altered miRNAs on predicted risk regions in variety of cancers. (PDF) [file pone.0096320.s005.pdf]

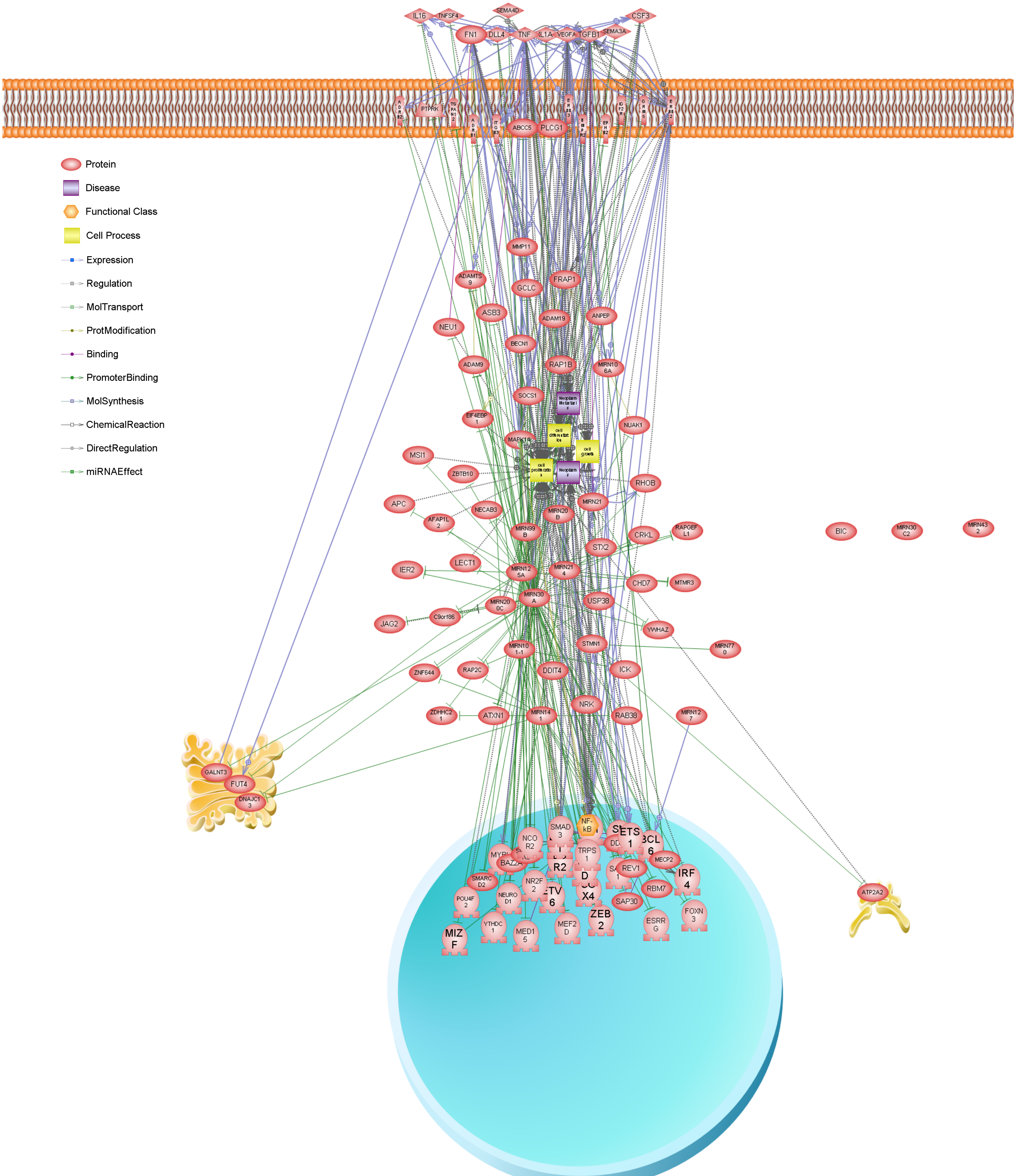


**Figure S5** Network of common altered microRNAs on predicted risk regions in variety of cancers. Network was constructed using pathway studio 9 software (shortest path algorithm). This network comprises 383 entities and 1121 relations and various type of transcription factors, protein kinases, small molecules, mrnas and mirnas serve as either validated or putative regulators.
