# Supplementary material for: Prediction of Potential Cancer-Risk Regions Based on Transcriptome Data: Towards a Comprehensive View
Source: PLoS One. 2014 May 5;9(5):e96320. doi: 10.1371/journal.pone.0096320 (PMC4010480; doi:10.1371/journal.pone.0096320)
Supplement: Table S9 — Expression patterns of common predicted regulators for altered microRNAs during different 11 cancers. (PDF) [file pone.0096320.s015.pdf]

Table S9 Expression patterns of common predicted regulators for altered microRNAs during different cancers (cancer data of 11 microarray studies: breast, colorectal, endometrial, gastric, liver, lung, ovarian, pancreatic, prostate and testicular cancers as well as glioblastoma). In total, 22 common regulators were predicted for different clusters of miRNAs which eight of them are located at PCSRs including YY1, SPIB, SOX10, NFIC, NR4A2, FOXD1, NFATC2 and HOXA5.

Gene symbol	Region	[a]PCSR	Breast	Endometrial	Ovarian	Prostate	Testicular	Colorectal	Liver	Gastric	Pancreatic	Lung	Glioblastoma
**GATA3**	chr10p14	-	↑	↑	↑		↑			↑	↑	↓	
**GATA2**	chr3q21.3	-		↓								↓	
**YY1**	chr14q34.2	✓			↓		↑					↑	
**ETS1**	chr11q23.3	-	↓	↑	↓		↓	↓				↓	
**MZF1**	chr19q13.43	-	↓	↓	↓	↓	↓					↓	
**FOXL1**	chr16q24.1	-		↑			↓			↑		↑	↓
**ELK1**	chrXp11.23	-			↑		↑	↑				↑	
**FOXO3**	Chr6q21	-	↓		↓		↓		↑	↓		↓	
**HOXA5**	chr7p15.2	✓	↓		↓				↑	↑		↓	↑
**SP1**	chr12q13.13	-		↓	↑		↑	↓				↑	↑
**AP1(JUN)**	chr1p32.1	-	↓	↓	↑		↓	↓				↓	
**SPIB**	chr19q13.33	✓			↑		↑	↓					
**ZNF354C**	5q35.3	-		↓			↑						
**SOX10**	chr22q13.1	✓			↑		↓	↓	↓	↓			
**USF1**	chr1q23.3	-		↑			↑		↑	↓			
**NFIC**	chr19p13.3	✓		↑	↓		↓		↓	↓		↓	
**NR4A2**	chr2q24.1	✓	↓	↓				↓	↓	↓		↓	
**SPI1**	chr11p11.2	-							↓			↓	
**FEV**	chr2q35	-			↑					↓			
**FOXD1**	chr5q13.2	✓			↑	↑	↓	↑		↑		↑	
**NFATC2**	chr20q13.2	✓	↓	↑	↑	↓	↓	↓		↑		↓	
**TFAP2A**	chr6p24.3	-	↑	↑	↑	↑	↑	↑	↑	↑	↑	↑	

[a] Predicted cancer-susceptibility region
