# Supplementary material for: Prediction of Potential Cancer-Risk Regions Based on Transcriptome Data: Towards a Comprehensive View
Source: PLoS One. 2014 May 5;9(5):e96320. doi: 10.1371/journal.pone.0096320 (PMC4010480; doi:10.1371/journal.pone.0096320)
Supplement: Table S10 — Details of raw CEL (microarray chip) expression data (obtained from GEO database) have been used in present study. (PDF) [file pone.0096320.s016.pdf]

Table S10 Details of raw CEL (microarray chip) expression data (obtained from GEO database) have been used in present study. This data are obtained from studies on breast, colorectal, endometrial, gastric, liver, lung, ovarian, pancreatic, prostate, testicular, bladder, intestine neuroendocrine, cervical and renal cancers as well as glioblastoma

	**Accession numbers**	**Cancer name**	**Study**
[a]Transcript microarray data	GSE17025	Endometrial cancer	Day *et al.*, 2011[1]
	GSE26910	Breast and prostate cancer	Planche *et al.*, 2011[2]
	GSE18520	Ovarian cancer	Mok et al., 2009[3]
	GSE15220	Testicular cancer	Cheung *et al.*, 2010[4]
	GSE29721	Liver cancer	Stefanska *et al.*, 2011[5]
	GSE19826	Gastric cancer	Wang *et al.*, 2012[6]
	GSE22780	Pancreatic adenocarcinoma	Balasenthil et al., 2010[7]
	GSE23878	Colorectal cancer	Uddin et al., 2011[8]
	GSE15824	Glioblastoma	Grzmil et al., 2011[9]
	GSE18842	Lung adenocarcinoma	Landi et al., 2008[10]
[a]MicroRNA microarray data	GSE23383	Ovarian cancer	Shahab et al., 2011[11]
	GSE23022	Prostate carcinoma	Wach et al., 2012[12]
	GSE39015	Cervical cancer	Hall et al., 2012[13]
	GSE41282	Renal cancer	Wach et al., 2012
	GSE39093	Bladder tumor	Xu et al., 2013[14]
	GSE33568	Intestinal neuroendocrine tumor	Li et al., 2013[15]

[a]We analyzed total of cancerous cel files vs. total of normal s in each study

1 Day, R. *et al.* Identifier mapping performance for integrating transcriptomics and proteomics experimental results. *BMC Bioinformatics* **12**, 213 (2011).
2 Planche, A. *et al.* Identification of Prognostic Molecular Features in the Reactive Stroma of Human Breast and Prostate Cancer. *PLoS One* **6**, e18640, doi:10.1371/journal.pone.0018640 (2011).
3 Mok, S. C. *et al.* A Gene Signature Predictive for Outcome in Advanced Ovarian Cancer Identifies a Survival Factor: Microfibril-Associated Glycoprotein 2. *Cancer Cell* **16**, 521-532, doi:10.1016/j.ccr.2009.10.018 (2009).
4 Cheung, H. H. *et al.* Genome-wide DNA methylation profiling reveals novel epigenetically regulated genes and non-coding RNAs in human testicular cancer. *Br J Cancer* **102**, 419-427, doi:http://www.nature.com/bjc/journal/v102/n2/suppinfo/6605505s1.html (2010).
5 Stefanska, B. *et al.* Definition of the Landscape of Promoter DNA Hypomethylation in Liver Cancer. *Cancer Research* **71**, 5891-5903, doi:10.1158/0008-5472.can-10-3823 (2011).
6 Wang, Q. *et al.* Upregulated INHBA expression is associated with poor survival in gastric cancer. *Medical Oncology* **29**, 77-83, doi:10.1007/s12032-010-9766-y (2012).
7 Balasenthil, S. *et al.* A Migration Signature and Plasma Biomarker Panel for Pancreatic Adenocarcinoma. *Cancer Prevention Research* **4**, 137-149, doi:10.1158/1940-6207.capr-10-0025 (2011).
8 Uddin, S. *et al.* Genome-Wide Expression Analysis of Middle Eastern Colorectal Cancer Reveals FOXM1 as a Novel Target for Cancer Therapy. *The American journal of pathology* **178**, 537-547 (2011).
9 Grzmil, M. *et al.* MAP Kinase-Interacting Kinase 1 Regulates SMAD2-Dependent TGF-β Signaling Pathway in Human Glioblastoma. *Cancer Research* **71**, 2392-2402, doi:10.1158/0008-5472.can-10-3112 (2011).
10 Landi, M. T. *et al.* Gene Expression Signature of Cigarette Smoking and Its Role in Lung Adenocarcinoma Development and Survival. *PLoS One* **3**, e1651, doi:10.1371/journal.pone.0001651 (2008).
11 Shahab, S. W. *et al.* Evidence for the Complexity of MicroRNA-Mediated Regulation in Ovarian Cancer: A Systems Approach. *PLoS One* **6**, e22508, doi:10.1371/journal.pone.0022508 (2011).
12 Wach, S. *et al.* MicroRNA profiles of prostate carcinoma detected by multiplatform microRNA screening. *International Journal of Cancer* **130**, 611-621, doi:10.1002/ijc.26064 (2012).
13 Hall, J. S. *et al.* Enhanced stability of microRNA expression facilitates classification of FFPE tumour samples exhibiting near total mRNA degradation. *Br J Cancer* **107**, 684-694, doi:http://www.nature.com/bjc/journal/v107/n4/suppinfo/bjc2012294s1.html (2012).
14 Xu, C. *et al.* miRNA-100 Inhibits Human Bladder Urothelial Carcinogenesis by Directly Targeting mTOR. *Molecular cancer therapeutics* **12**, 207-219 (2013).
15 Li, S.-C. *et al.* Global microRNA profiling of well-differentiated small intestinal neuroendocrine tumors. *Mod Pathol*, doi:http://www.nature.com/modpathol/journal/vaop/ncurrent/suppinfo/modpathol2012216s1.html (2013).
